# Regional deep atrophy: Using temporal information to automatically identify regions associated with Alzheimer’s disease progression from longitudinal MRI

**DOI:** 10.1162/imag_a_00294

**Published:** 2024-09-18

**Authors:** Mengjin Dong, Long Xie, Sandhitsu R. Das, Jiancong Wang, Laura E.M. Wisse, Robin deFlores, David A. Wolk, Paul A. Yushkevich

**Affiliations:** University of Pennsylvania Perelman School of Medicine, Philadelphia, PA, United States; Siemens Healthineers, Malvern, PA, United States; Lund University, Lund, Sweden; Institut National de la Santé et de la Recherche Médicale (INSERM), Caen, France

**Keywords:** Alzheimer’s disease, longitudinal analysis, structural MRI, attention maps, medial temporal lobe, sensitive biomarkers

## Abstract

Longitudinal assessment of brain atrophy, particularly in the hippocampus, is a well-studied biomarker for neurodegenerative diseases, such as Alzheimer’s disease (AD). Estimating brain progression patterns can be applied to understanding the therapeutic effects of amyloid-clearing drugs in research and detecting the earliest sign of accelerated atrophy in clinical settings. However, most state-of-the-art measurements calculate changes directly by segmentation and/or deformable registration of MRI images, and may misreport head motion or MRI artifacts as neurodegeneration, impacting their accuracy. In our previous study, we developed a deep learning method DeepAtrophy that uses a convolutional neural network to quantify differences between longitudinal MRI scan pairs that are associated with time. DeepAtrophy has high accuracy in inferring temporal information from longitudinal MRI scans, such as temporal order or relative interscan interval. DeepAtrophy also provides an overall atrophy score that was shown to perform well as a potential biomarker of disease progression and treatment efficacy. However, DeepAtrophy is not interpretable, and it is unclear what changes in the MRI contribute to progression measurements. In this paper, we propose Regional Deep Atrophy (RDA), which combines the temporal inference approach from DeepAtrophy with a deformable registration neural network and attention mechanism that highlights regions in the MRI image where longitudinal changes are contributing to temporal inference. RDA has similar prediction accuracy as DeepAtrophy, but its additional interpretability makes it more acceptable for use in clinical settings, and may lead to more sensitive biomarkers for disease monitoring and progression understanding in preclinical AD.

## 1 Introduction

Long before symptoms manifest, individuals afflicted with Alzheimer’s disease (AD) experience accelerated neuronal degeneration compared with their unaffected counterparts of similar age. This rapid neuronal decline typically initiates in the medial temporal lobe (MTL) region, and gradually extends throughout the entire brain (Braak & Braak, 1995;Giannakopoulos et al., 2003;Gunten et al., 2006). Clinical manifestations such as cognitive impairment or memory loss typically arise in the later stages of AD. Atypical variants of AD are also observed, such as posterior cortical atrophy (PCA) (Crutch et al., 2012), primarily affecting the back of the brain, and logopenic variant primary progressive aphasia (lvPPA), primarily impacting language-associated regions (Ossenkoppele et al., 2015). AD pathology is frequently accompanied by additional proteinopathies (TDP-43 pathology, alpha-synucleinopathy) and cerebrovascular disease, which contribute to neurodegeneration and cognitive decline. Hence, patterns of neurodegeneration in AD are complex and driven by multiple factors. Recent successful clinical trials of antiamyloid agents donanemab (Sims et al., 2023) and lecanemab (Van Dyck et al., 2023) showed strongest treatment effects in participants at earlier AD stages, suggesting that it is best to intervene in the disease process at its nascent stage, before significant neuronal damage ensues and cognitive symptoms develop. However, intervention in presymptomatic and early symptomatic stages of AD requires highly sensitive biomarkers to monitor disease progression and treatment efficacy.

Measurement of volumetric change in the hippocampus and entorhinal cortex (both part of the MTL) on longitudinal structural MRI (sMRI) is a well-established biomarker for tracking disease progression in AD (Holland et al., 2009;Sperling, Aisen, et al., 2011). Although the aducanumab (Budd Haeberlein et al., 2022) and donanemab (Sims et al., 2023) clinical trials have reported paradoxical longitudinal sMRI findings, with greater whole brain volume decrease in the treatment group, the hippocampus did not follow this trend, with the placebo group exhibiting greater or similar hippocampal atrophy (Budd Haeberlein et al., 2022;Sims et al., 2023) compared with the treatment group. Nevertheless, the paradoxical brain volume findings dampen the perspective of longitudinal sMRI serving as an outcome measure in future trials. However, longitudinal sMRI remains a crucial tool in AD research. Compared with cognitive assessments, biomarkers derived from longitudinal structural MRI have proven to be more sensitive indicators to disease progression in early AD, as evidenced by previous studies comparing longitudinal MRI and cognitive metrics among groups with varying degrees of AD severity and cognitively intact controls (Caroli & Frisoni, 2010;Hua et al., 2009). Compared with cross-sectional sMRI measures, longitudinal sMRI measures are less affected by interindividual variability in brain development and show stronger association with AD pathology (Xie et al., 2022). Longitudinal sMRI will likely play a key role in understanding the causes of paradoxical brain volume findings reported above, including understanding the contribution of ARIA (Hampel et al., 2023;Hua et al., 2009;Schuff et al., 2009;Sperling, Jack, et al., 2011) to these findings. Longitudinal sMRI will also be a key tool for long-term study of the effects of new antiamyloid treatments on the brain, given the greater availability and lower cost of MRI compared with PET.

Multiple methods have been developed to measure longitudinal volume changes of brain structures. A natural way to measure longitudinal volume change is to quantify volumes in each scan from the same subject independently and compare the volume difference. However, this method turns out to be susceptible to small errors in each measurement and generates change measurements with large variance (Das et al., 2012;Hua et al., 2008;Schuff et al., 2009). The alternative is to analyze pairs of longitudinal MRI scans jointly and quantify change in volume directly, typically via deformable registration. In a recent grand challenge (Cash et al., 2015), there were two dominant approaches to longitudinal volume change analysis. One class of methods is*deformation-based morphometry (DBM)*, also known as*tensor-based morphometry (TBM)*(Das et al., 2012;Hua et al., 2008;Reuter et al., 2012;Xie, Wang, et al., 2019). In DBM, deformable registration is applied to a pair of longitudinal MRI scans, and the deformation field capturing the spatial transformation from the*baseline image*(term used to refer to the first scan in a longitudinal study) to the*follow-up image*(term used to refer to later scans in a longitudinal study) at each voxel is obtained. The hippocampus (or another structure of interest) is extracted in the baseline image, and the deformation field is applied to this segmentation to infer the change in hippocampal volume over time. The other class of approaches is*Boundary Shift Integral (BSI)*(Gunter et al., 2003;Leung et al., 2009;Prados et al., 2015). Rather than calculating a voxel-wise deformation field in DBM, BSI uses intensity information along the boundaries of the hippocampus (or other structures of interest) to infer boundary displacements or shifts between the baseline and follow-up images, from which volumetric change is estimated. Critically, both DBM and BSI implicitly assume that any deformation detected between a pair of longitudinal sMRI scans is attributable to neurodegeneration, whereas in practice, measured deformations may also be caused by many factors such as motion, imaging artifacts, variation in head position, scanner/protocol differences, participant hydration, and transient effects related to treatment, such as transient ARIA (Hampel et al., 2023), which can confound the results of atrophy estimation.Table 1summarizes the factors that may be detected as volume change in DBM analysis and categorizes them as systematic with respect to time versus nonsystematic.

**Table 1.** Changes observed in longitudinal MRIs can be categorized into systematic and nonsystematic, and may arise from various factors including biological, nonbiological, and treatment-induced influences.

	Systematic (detected by RDA)	Nonsystematic
Biological	Neurodegeneration	Head motionVariation in hydration levels
Nonbiological	Scanner changes/protocol updates	MRI artifacts, noise
Treatment-induced	Chronic ARIA	Transient ARIA

Notably, nonsystematic factors such as head motion, individual hydration status, MRI artifacts, and transient amyloid-related imaging abnormalities (ARIA) can be independent of time, whereas systematic changes such as atrophy and scanner upgrades are time dependent.

Sankar et al. (2017)evaluated hippocampal progression estimated from five distinct state-of-the-art DBM algorithms in ADNI and reported that in 20% to 40% of the cases these algorithms detected an increase in hippocampal volume over time, an unexpected findings for this population. Moreover, there was very little overlap between the algorithms in terms of the subjects for which they detected hippocampal growth, suggesting that the findings of growth were erroneous. The findings ofSankar et al. (2017)are consistent with the hypothesis that some of the nonsystematic and nonbiological factors listed inTable 1contribute to errors in the hippocampal atrophy measures detected by DBM methods. Recent image registration methods based on deep learning (Dalca et al., 2019;Fu et al., 2020;Mok & Chung, 2020) show results comparable with conventional optimization-based registration methods, while significantly reducing inference time, and are hence an attractive alternative for quantification of longitudinal changes in MRI. However, deep learning-based registration models are typically trained using the same objective functions as those optimized in conventional registration methods, that is, to minimize image dissimilarity while regularizing the transformation field. Therefore, simply applying deep learning-based registration models in the context of DBM is unlikely to help disambiguate neurodegeneration from confounding factors.

To partially address the confounds associated with extraneous factors in longitudinal brain atrophy quantification, we recently developed the deep learning algorithm*DeepAtrophy*(Dong et al., 2021). Specifically, DeepAtrophy addresses confounds that are nonsystematic with respect to time, that is, random head motion, MRI artifacts, and transient biological or treatment-induced factors. DeepAtrophy used deep learning methods to infer a relative progression rate from a pair of longitudinal MRI scans, which is compared with the progression rate of normal aging group. Previous studies show that disease groups tend to exhibit more progressive changes (atrophy) than healthy control groups over the same period. However, DeepAtrophy’s prediction of relative interscan intervals assumes a uniform rate of progression for all subjects, regardless of their disease status. As a result, if an individual experiences accelerated brain atrophy, such as in the case of an AD patient, the predicted interscan interval is expected to be longer than actual interscan intervals. The inference of DeepAtrophy is conducted by two auxiliary objectives used when training deep learning models: for a pair of images input to the model in an arbitrary order, determine the correct scan temporal order (STO); and for two pairs of images with the scan time interval of one pair strictly inside the other pair, also arbitrarily input to the model, determine which pair has a longer relative interscan interval (RISI). In other words, when training the DeepAtrophy model, we input sets of scans of the same individual in an arbitrary order and ask the model to infer information about time order based on image content. Since neurodegeneration is associated with the passage of time, while other factors, such as imaging artifacts, are more likely to be independent of time, DeepAtrophy is likely implicitly detecting neurodegeneration by learning to correctly infer temporal information from longitudinal MRI scans. Our evaluation reported that DeepAtrophy detects hippocampal “growth” in a substantially smaller proportion of longitudinal MRI scan pairs from the Alzheimer’s disease Neuroimaging Initiative (ADNI) than ALOHA, a leading DBM-based pipeline (Das et al., 2012) (11.5% vs. 24.5%). Significantly, DeepAtrophy detects an accelerated progression rate in a preclinical AD group (i.e., cognitively unimpaired individuals with evidence of AD pathology based on a positive β-amyloid positron emission tomography (PET) scan, A+ CU) compared with a control group of β-amyloid PET negative cognitively unimpaired individuals (A- CU), using scans within only 2 years of baseline for each individual, demonstrating sensitivity to subtle changes in longitudinal MRI scans. However, the downside of DeepAtrophy is that it only generates a scalar relative progression score for each pair of longitudinal MRI scans, a summary measure that cannot be directly attributed to atrophy in any specific anatomical structure or region. This is a significant limitation, since in clinical applications and/or clinical trials, model interpretability and transparency are important considerations; the current paper addresses this limitation. Another limitation of DeepAtrophy is that it cannot distinguish neurodegeneration from other systematic factors inTable 1(i.e., scanner change or chronic ARIA), and those factors must be controlled for in study design to the extent possible.

To our knowledge, only a few papers besides DeepAtrophy use deep learning and self-supervised learning methods to extract features that can explicitly represent structural progression of AD from MRI images. InOuyang et al. (2022), the authors used self-supervised learning methods to constrain over the embedding vector space of longitudinal image pairs, and generate embedding vectors representative of progression related to aging. InRen et al. (2022), a longitudinally consistent spatiotemporal representation learning framework was proposed for longitudinal segmentation and spatial temporal consistency evaluation. However, both studies were based on downsampled whole brain images, as opposed to our approach, which explicitly focuses on the MTL, a region of the brain where earliest AD-related neurodegeneration occurs, and which attempts to isolate changes in longitudinal MRI that are truly driven by neurodegeneration, rather than unrelated factors.Rahim et al. (2023)proposed a deep learning model to predict AD conversion within 3 years and used guided GradCam to interpret model predictions across the whole brain. Their model achieved high classification accuracy by incorporating clinical scores; however, the highlighted regions identified by the model showed limited alignment with known AD progression patterns.

In this paper, we propose a new method called Regional Deep Atrophy (RDA). Like DeepAtrophy, RDA is trained using loss functions that leverage temporal information to teach the network to distinguish between systematic and nonsystematic changes. However, unlike DeepAtrophy, which functions as a “black box” producing a single measure of progressive change and lacking interpretability, RDA is crafted to emulate a conventional DBM approach. In RDA, a deformation field is computed between pairs of scans, and the longitudinal change measure is derived from applying this deformation field to regions of interest (ROIs). The deformation field can be generated by any image registration algorithm and remains fixed during RDA training. However, instead of focusing solely on a predefined structure like the hippocampus, RDA employs an attention mechanism to identify ROIs in each scan pair where deformation is linked to systematic changes. This attention model utilizes a segmentation-like network, specifically a 3D U-Net, to generate ROIs associated with systematic shrinkage (characteristic of gray matter structures) and expansion (characteristic of areas filled with cerebrospinal fluid). The underlying expectation is that areas of the image most affected by nonsystematic factors, such as head motion, would be excluded from the shrinkage/expansion ROIs, which would in turn enhance the overall sensitivity of the RDA change measures to neurodegeneration.

Due to the additional constraints in the RDA design compared with the “black box” DeepAtrophy model, we hypothesized that RDA would perform slightly worse than DeepAtrophy in terms of temporal order metrics (analogous to the proportion of “hippocampus growers” inSankar et al., 2017), but that it would still outperform conventional DBM on these metrics. Similarly, we hypothesized that RDA-derived change measures would track AD stage and show association with AD biomarkers more strongly than DBM-based measures, and similarly to DeepAtrophy-derived measures. Lastly, we hypothesized that the shrinkage and expansion saliency maps constructed by coregistration and averaging of RDA attention maps would reflect known topography of AD neurodegeneration, with shrinkage in the MTL gray matter and expansion in the ventricles. We test these hypotheses using a large longitudinal MRI dataset from ADNI (Mueller et al., 2005).

## 2 Material and Methods

The overall structure of the RDA network is shown inFigure 1. RDA integrates the deformation field generated by the DBM method ALOHA over shrinking and expansion ROIs generated by the attention network to generate summary measures of shrinking and expansion. During training, longitudinal scans from the same individual are input in arbitrary time order to optimize STO and RISI loss functions previously proposed in our DeepAtrophy work (Dong et al., 2021). The Scan Temporal Order (STO) loss tests whether two longitudinal scans are input in correct or reverse temporal order. The Relative Interscan Interval (RISI) loss classifies the ratio of interscan intervals between two pairs of scans (e.g., one pair 1 year apart and another pair 3 years apart) into a set of discrete categories. The RISI loss requires two pairs of scans to be input at a time, each pair processed by a separate copy of the attention network. During inference, one pair of scans is input to the attention network in correct time order and the summary shrinking/expansion measures are used for subsequent analysis.

**Fig. 1.**
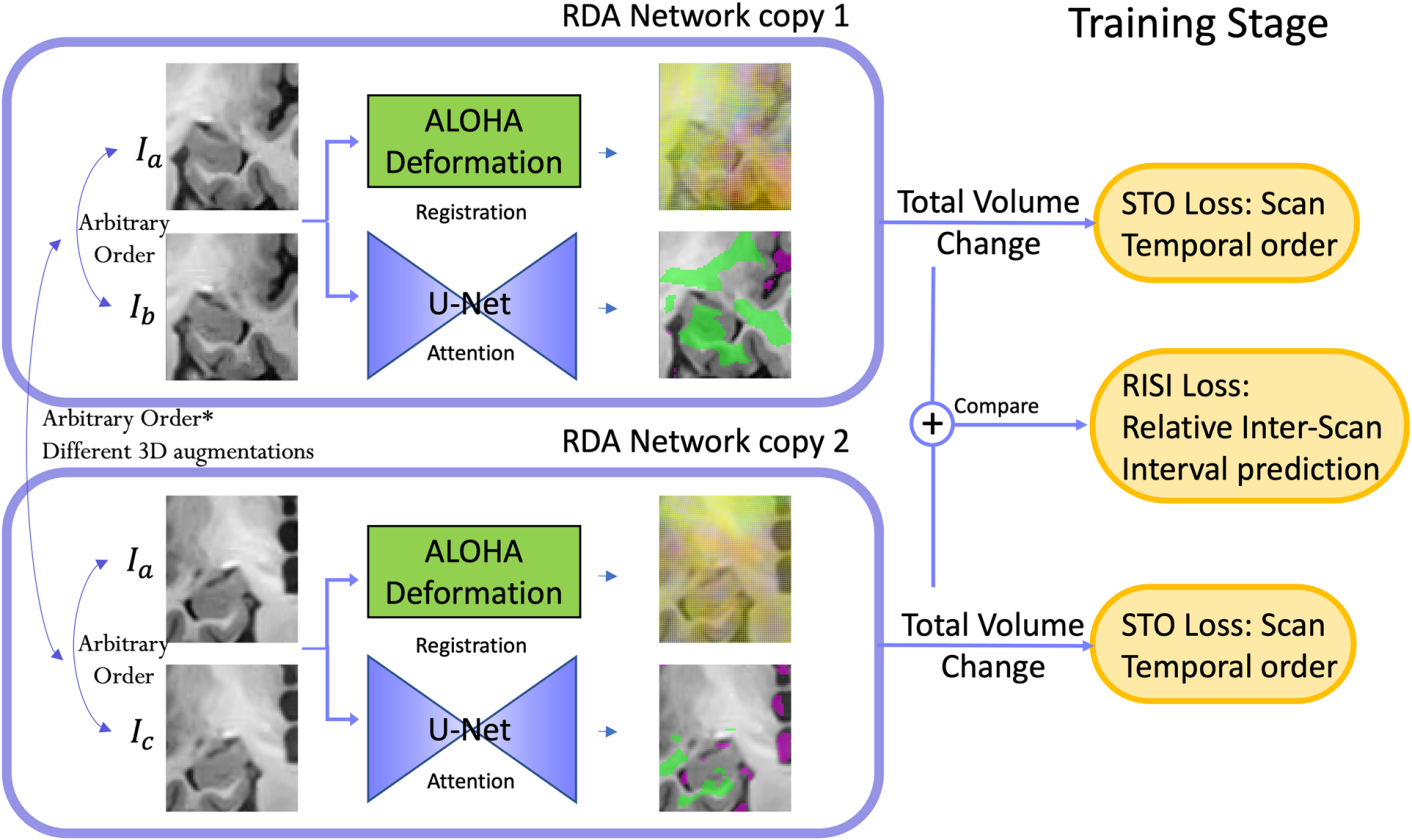

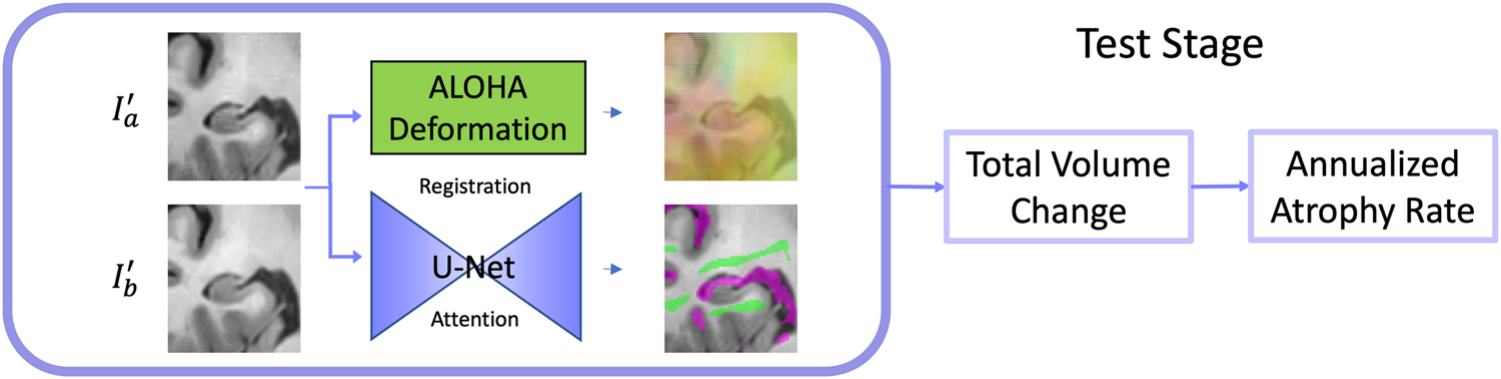
The overall architecture of the Regional Deep Atrophy (RDA) pipeline involves two pairs of images of the same subject (pairs$\left(I_{a},I_{b}\right)$and$\left(I_{a},I_{c}\right)$in this example), which are input to two copies of the RDA network in an arbitrary order. For each copy, the image pair is fed into a U-net-like Attention Network. Subsequently, a change in volume is computed from the deformation field generated by ALOHA for each image pair, alongside the shrinkage/expansion attention maps produced by the Attention Network. In these maps, the green area represents the shrinkage region, while the purple area represents the expansion region. The Scan Temporal Order (STO) loss is computed based on whether the sign of the total volume change aligns with the ordering of the input image pair, and the Relative Interscan Interval (RISI) loss is determined by whether a larger total volume change corresponds to a longer interscan interval between two image pairs. During the testing stage, only a longitudinal image pair is needed, and the total volume change is directly calculated as the final output for this pair, without STO or RISI loss calculation.

The structure of this section is as follows:Section 2.1introduces the dataset and preprocessing procedures utilized in our experiments;Section 2.2provides a brief introduction of a DBM method, ALOHA;Section 2.3elaborates on the design of the RDA network and describes how STO and RISI losses are implemented for RDA;Section 2.4introduces the evaluation metrics employed in this study and outlines the algorithms used for comparison; finally,Section 2.5specifies some hyperparameters and implementation details.

### 2.1 Participants and preprocessing

Data used in this study were obtained from the Alzheimer’s Disease Neuroimaging Initiative (ADNI,adni.loni.usc.edu). The ADNI was launched in 2003 as a public–private partnership, led by Principal Investigator Michael W. Weiner, MD. The primary goal of ADNI has been to test whether serial magnetic resonance imaging (MRI), positron emission tomography (PET), other biological markers, and clinical and neuropsychological assessment can be combined to measure the progression of mild cognitive impairment and early Alzheimer’s disease. For up-to-date information, seewww.adni-info.org.

In total, 502 participants from ADNI2 and ADNI GO were included in this study. Informed consent was obtained from all participants for being included in the study. All participants have 2-6 T1-weighted MRI scans, spanning from 0.25 to 6 years from baseline. Detailed MRI acquisition information can be found inSupplementary Section S3. In total, 4,927 pairs of same-subject MRI scans are included in this dataset. A summary standardized uptake value ratio (SUVR) derived from associated Florbetapir PET images collected within 0.5 years of the baseline MRI scan was used to determine β-amyloid (A) status (threshold of 1.11;Landau et al., 2012). Participants were grouped into four cohorts corresponding to progressive stages along the AD continuum: A- cognitively unimpaired older adults (A-CU), A+ cognitively unimpaired older adults (A+ CU, also referred to as the*preclinical AD*group), and A+ early and late Mild Cognitive Impairment (A+ eMCI and A+ lMCI).

Participants were divided into training (n = 155), validation (n = 21), and test (n = 326) groups. In total, 72 subjects with only 2 MRI scans were assigned to the test group since in the training stage at least three scans are required. All 85 preclinical AD subjects (A+ CU) were also assigned to the test group to keep a large enough sample size for the group difference analysis. The rest of the subjects (347) were split into training, validation, and test groups by a proportion of 45%, 5%, and 50% for each diagnosis group. The numbers were rounded to favor the evaluation group for each diagnostic group. Demographics such as age, sex, and Mini-Mental State Examination (MMSE) scores of all four groups in the training and test sets are included inTable 2.

**Table 2.** Characteristics of the selected ADNI2/GO participants whose T1 MRI scans were used for the Regional Deep Atrophy (RDA) and comparison experiments for this paper.

	Training set (n = 155)				Test set (n = 326)			
	A- CU (n = 64)	A+ CU (n = 0)	A+ eMCI (n = 49)	A+ lMCI (n = 42)	A- CU (n = 103)	A+ CU (n = 85)	A+ eMCI (n = 79)	A+ lMCI (n = 59)
Age (years old)	71.5 (5.7)	-	73.3 (7.4)	71.9 (7.2)	72.4 (6.3)	75.2 (5.8) **	74.0 (6.9)	72.5 (6.5)
Sex	27F (42%) 37M	-	16F (33%) 33M	21F (50%) 21M	56F (54%) 47M	58F (68%) 27M	39F (49%) 40M	28F (47%) 31M
Edu (years)	17.4 (2.3)	-	16.2 (3.0) *	16.8 (2.7)	16.8 (2.4)	16.0 (2.7)	15.3 (2.9) ***	16.4 (2.7)
MMSE	29.1 (1.2)	-	28.0 (1.5)	26.7 (1.8)	29.1 (1.2)	29.0 (1.1)	28.1 (1.6)	27.3 (1.9)
#Scans	4.64 (1.24)	-	5.13 (1.17)	5.09 (0.93)	4.35 (1.47)	4.00 (1.52)	4.81 (1.45)	4.51 (1.33)
#Interval (years)	2.74 (1.18)	-	2.75 (1.44)	2.75 (1.29)	2.62 (1.31)	2.45 (1.22)	2.65 (1.41)	2.12 (1.47)

All subjects in the training and test sets had 2–6 scans between 0.25 and 6 years from the baseline.

*Notes*: All statistics in the training and test sets are in comparison with the corresponding A- CU group. Standard Deviation is reported in parentheses. Independent two‐sample*t*‐test (continuous variables with normal distribution, for age and education) and contingency*χ*^2^test (sex) were performed.

* p < 0.05; **p < 0.01; ***p < 0.001; ****p < 0.0001.

n = number of subjects; A+/A-: β-amyloid positive/negative; CU = cognitively unimpaired adults; eMCI = early mild cognitive impairment; lMCI = late mild cognitive impairment; Edu = years of education; MMSE = minimental state examination.

The same preprocessing steps as in DeepAtrophy (Dong et al., 2021) were applied to our dataset. First, the ADNI T1-MRI scans were up-sampled by a factor of two in the coronal plane using nonlocal mean super-resolution technique (Coupé et al., 2013;Manjón et al., 2010). Next, the left and right MTLs were segmented using ASHS-T1 segmentation software (Xie, Wisse, et al., 2019) and image regions around the left and right MTLs were cropped with a margin of ~10 voxels. These left and right MTL images were treated separately in subsequent analysis, and the MTL segmentations were not used in the RDA model other than for cropping the MTL region. However, ASHS-T1 MTL segmentations were used by the DBM techniques for comparison. Next, rigid alignment was performed separately for the left and right MTL regions in each pair of longitudinal scans. The rigid transformation was decomposed into two equal halves (i.e.,$R=R_{half}\circ R_{half}$), and these halves were applied to the fixed and moving images, so that the amount of aliasing after applying the rigid transformation would be equivalent for the fixed and moving images (Yushkevich et al., 2010). Each pair of scans was registered twice, with the earlier scan as the fixed image and the later scan as the moving image, and vice versa. Overall, 19,708 pairs of 3D images were analyzed (4,927 scan pairs * 2 sides * 2 registration directions). Since manually checking this number of image pairs for registration errors is impractical, we computed the Structural SIMilarity (SSIM) score (Wang et al., 2004) between registered scan pairs and excluded all scan pairs with SSIM <0.6. Visual examination of scan pairs shows that the SSIM score of most successfully registered 3D scan pairs is between 0.7 and 0.9, and an SSIM score <0.6 of an image pair would mean large misalignment between two images. A total of 1,414 scan pairs (7.2%) were rejected through this procedure.

### 2.2 Image registration between longitudinal pairs using ALOHA

In our model, we first obtain local deformation fields between longitudinal image pairs using the DBM pipeline Automatic Longitudinal Hippocampal Atrophy (ALOHA) (Das et al., 2012), a method shown to be competitive for hippocampus atrophy estimation as evaluated byCash et al. (2015). These deformation fields capture changes in anatomy in interpretable terms; for instance, the determinant of the Jacobian matrix of the deformation field provides a local measure of tissue shrinkage or expansion over time. ALOHA is specifically designed for evaluating longitudinal changes in the brain by registering the baseline and follow-up images of the same individual to a middle space, thus performing a symmetric registration. This approach avoids a particular type of bias resulting from an uneven number or amount of interpolation for the baseline and follow-up images in one-directional image registration.

Deformation fields calculated from ALOHA pipeline are saved along with pairs of same-subject scans for RDA analysis. In the atrophy estimation process of the ALOHA pipeline, these deformation fields are applied to a mesh derived from the ASHS-T1 hippocampus segmentation (Xie et al., 2019) of the fixed image to estimate the hippocampus volume in the moving image. The annualized rate of change in hippocampus volume is then derived in ALOHA by calculating the difference between fixed and moving hippocampus volumes, dividing it by the fixed hippocampus volume, and dividing further by the interscan interval. The estimation of annualized rate of change when multiple pairs of scans are available for a subject is discussed inSection 2.4.

### 2.3 RDA model architecture: Attention model and time-related losses

The attention map in RDA is implemented as a 3D U-Net (Çiçek et al., 2016;Ronneberger et al., 2015) and functions similar to a 3D image segmentation network. It takes fixed and moving MTL ROIs as inputs and produces three activation maps corresponding to expansion, shrinkage, and background regions. We model shrinkage and expansion separately because the MTL region contains structures that shrink with aging and disease progression (e.g., hippocampus, amygdala, cortex, white matter) as well as structures that increase in volume (e.g., ventricles). The background region corresponds to portions of the image where there are no changes or where the deformation field mainly reflects nonsystematic changes, such as those caused by MRI artifacts. These activation maps are converted to “almost binary” masks using a SoftMax layer with a sufficiently large temperature coefficient (τ=100).

Let$S_{SHR}^{j,t_{1},t_{2}}$and$S_{EXP}^{j,t_{1},t_{2}}$describe the outputs of the SoftMax layer corresponding to shrinking and expansion for a subjectjwith scans at timepoints$t_{1}$and$t_{2}$, where the scan at time$t_{1}$is used as the fixed image for deformable registration, and$t_{2}$as the moving image. Let the corresponding deformation field generated by ALOHA be denoted$\Phi^{j,t_{1},t_{2}}.$. To determine the change in volume induced by$\Phi^{j,t_{1},t_{2}}$on the shrinkage and expansion regions, we compute the volumes of these regions in the fixed image, denoted as$V_{SRK,1}^{j,t_{1},t_{2}}$,$V_{EXP,1}^{j,t_{1},t_{2}}$, and the moving image, denoted as$V_{SRK,2}^{j,t_{1},t_{2}}$,$V_{EXP,2}^{j,t_{1},t_{2}}$. The volumes of the shrinkage and expansion ROIs in the fixed image are:

$$\begin{array}{c} V_{SHR,1}^{j,t_{1},t_{2}}=\int S_{SHR}^{j,t_{1},t_{2}}(\text{x})dx\;V_{EXP,1}^{j,t_{1},t_{2}}=\int S_{EXP}^{j,t_{1},t_{2}}(\text{x})dx \end{array}$$ (1)

and the corresponding volumes in the moving image are:

$$\begin{array}{c} \begin{array}{l} V_{SHR,2}^{j,t_{1},t_{2}}=\int \Phi^{j,t_{1},t_{2}-1}\left(S_{SHR}^{j,t_{1},t_{2}}(\text{x})\right)dx\\ V_{EXP,2}^{j,t_{1},t_{2}}=\int \Phi^{j,t_{1},t_{2}-1}\left(S_{EXP}^{j,t_{1},t_{2}}(\text{x})\right)d\text{x}. \end{array} \end{array}$$ (2)

It is important to note that when a scan pair is processed in a reversed orderj,$t_{2}$,$t_{1}$, the deformation field$\Phi^{j,t_{2},t_{1}}$is calculated using the scan at time$t_{2}$as the fixed image and$t_{1}$as the moving image. This results in two independent image registration tasks for the two orderings of the scan pair, and the resulting deformation field$\Phi^{j,t_{2},t_{1}}$are not the same as$\Phi^{j,t_{1},t_{2}-1}$. Consequently, the volumes$V_{SHR,1}^{j,t_{1},t_{2}}$and$V_{SHR,2}^{j,t_{2},t_{1}}$are not necessarily equal, because the attention network will produce different outputs when inputs are reordered; the same applies to the expansion regions. This necessitates the use of the somewhat cumbersome notation above to represent ROI volumes in moving and fixed image space.

The absolute change in volume induced by the deformation$\Phi^{j,t_{1},t_{2}}$on the shrinkage/expansion ROIlis defined as

$$\begin{array}{c} A_{SHR}^{j,t_{1},t_{2}}=V_{SHR,2}^{j,t_{1},t_{2}}-V_{SHR,1}^{j,t_{1},t_{2}} \end{array}$$ (3)

$$\begin{array}{c} A_{EXP}^{j,t_{1},t_{2}}=V_{EXP,2}^{j,t_{1},t_{2}}-V_{EXP,1}^{j,t_{1},t_{2}}. \end{array}$$ (4)

Under the assumption that in the brains of older adults the volume of gray and white matter structures decreases over time while the volume of the ventricles increases over time, we expect$A_{SHR}^{j,t_{1},t_{2}}$to be negative and$A_{EXP}^{j,t_{1},t_{2}}$to be positive when scans are input in correct temporal order, that is,$t_{2}>t_{1}$, and we expect the opposite when the scans are input in inverse temporal order.

Training the RDA network involves minimizing the weighted sum of two classification losses,*scan temporal order (STO)*loss and*relative interscan interval (RISI) loss*, with respect to the parameters of the attention network. These losses were first introduced in our DeepAtrophy work (Dong et al., 2021) and are reused here with a slight modification to the RISI loss to make the update of model parameters more efficient. The absolute measures of expansion and shrinkage$A_{SHR}^{j,t_{1},t_{2}}$and$A_{EXP}^{j,t_{1},t_{2}}$serve as inputs to the STO and RISI losses.

The STO loss encapsulates the expectation that brain atrophy is monotonic in time. It is implemented as a classification loss that infers whether two scans are input in correct temporal order or in reverse order. For STO loss calculation, let$y^{j,t_{1},t_{2}}=\text{sign}({\text{t}}_{2}\;\;-{\text{t}}_{1})$represent the ground truth temporal order of the image pair (1 if correct, -1 if reversed). The STO loss for one image pair is implemented as the cross-entropy loss:

lSTO1  =lSTO1SRK  +lSTO1EXP  =lCE([−ASHRj,t1,t2,ASHRj,t1,t2];yj,t1,t2)     + lCE([AEXPj,t1,t2,−AEXPj,t1,t2];yj,t1,t2). (5)

The RISI loss encapsulates the expectation that the magnitude of change in brain structures measured from two MRI images of the same individual,$I^{j,t_{1}}$and$I^{j,t_{2}}$should be approximately proportional to the time interval between the scans$\left(t_{2}\;\;-t_{1}\right)$(unit: days), that is,

$$change(I^{j,t_{1}},I^{j,t_{2}})∼\Delta t_{12}\;\;where\;\;\;\;\Delta t_{12}=t_{2}-t_{1}.$$ (6)

The key observation in the design of the RISI loss is that the constant of proportionality in the relationship above is different for every individual and is unknown when training the network. Therefore, we cannot formulate this loss as simply predicting the length of the interscan interval from a pair of scans. Instead, the loss is formulated in relative terms, where two pairs of scans$\left(t_{1},t_{2}\right)$and$(t_{3},t_{4})$from the same individualjare compared, and the objective is to infer information about the relative lengths of the interscan intervals$\left(t_{2}-t_{1}\right)$and$\left(t_{4}-t_{3}\right)$.

As in DeepAtrophy, training the network with the RISI loss involves using two identical copies, or branches, of the RDA network with shared weights (seeFig. 1). The selection of two pairs of images is based on ensuring that the scan times of one pair strictly fall within the scan times of the other pair. Formally, we require$\left[\text{min(}t_{1},\;t_{2}\text{)},\;\max(t_{1},\;t_{2})\right]\subseteq \left[\text{min}(t_{3},\;t_{4}),\;\max(t_{3},\;t_{4})\right]$, if$\left(t_{4}\;\;-t_{3}\right)$>$\left(t_{2}\;\;-t_{1}\right)$or$\left[\text{min}(t_{3},\;t_{4}),\;\max(t_{3},\;t_{4})\right]\subseteq [\text{min}(t_{1},\;t_{2}),$$\max(t_{1},\;t_{2})]$if$\left(t_{4}\;\;-t_{3}\right)$<$\left(t_{2}\;\;-t_{1}\right)$. Furthermore, we allow for one, but not both, endpoint of the interscan intervals to coincide. This means that the two pairs of scans may consist of three or four distinct scans, ensuring that there is enough temporal separation between the two pairs. The ordering between$\left(t_{1},t_{2}\right)$and$\left(t_{3},t_{4}\right)$can be arbitrary. In this two-branch design, the STO loss is computed separately for each branch using (5) and summed to obtain the total STO loss for the two scan pairs.

For RISI calculation, under the assumption that the extent of progressive changes in brain structures is proportional to time, we anticipate the following relationships for the shrinkage and expansion areas identified by the attention network:

$$\begin{array}{l} \frac{A_{SHR}^{j,t_{1},t_{2}}}{A_{SHR}^{j,t_{3}t_{4}}}=\frac{t_{2}-t_{1}}{t_{4}-t_{3}},\\ \frac{A_{EXP}^{j,t_{1},t_{2}}}{A_{EXP}^{j,t_{3},t_{4}}}=\frac{t_{2}-t_{1}}{t_{4}-t_{3}}. \end{array}$$ (7)

While it is theoretically feasible to employ a regression loss to train the network to adhere to this relationship, in practice, we encountered poor convergence with such an approach (observed in both DeepAtrophy and RDA training). Instead, consistent with methods utilized in order learning (Frank & Hall, 2001;L. Li & Lin, 2007), this regression is transformed into a multiclass classification problem. Rather than directly predicting the exact interscan interval ratio, we predict the absolute value of ground-truth interscan interval ratio$\frac{\left|t_{2}-t_{1}\right|}{\left|t_{4}-t_{3}\right|}$, which is then categorized into four classes representing the ranges [0, 0.5], [0.5, 1], [1, 2], and [2, +∞].

To formulate RISI as a categorical loss, we establish a differentiable mapping between the absolute volume change values,$A_{l}^{j,t_{1},t_{2}}$and$A_{l}^{j,t_{3},t_{4}}$, and a one-hot vector of probability values corresponding to the four ranges above. We use the symbollto represent the type of region (EXP/SRK) in the formulas below, which are analogous for both types of regions.

Let us represent the pair of absolute change values$A_{l}^{j,t_{1},t_{2}}$and$A_{l}^{j,t_{3},t_{4}}$as a point in${\mathbb{R}}^{2}$. Then the equation$\frac{\left|A_{l}^{j,t_{1},t_{2}}\right|}{\left|A_{l}^{j,t_{3},t_{4}}\right|}=const$corresponds to a line passing through the origin. The four ranges above then correspond to cone-like regions of${\mathbb{R}}^{2}$separated by such lines, as depicted inFigure 2. For instance, if$\frac{\left|A_{l}^{j,t_{1},t_{2}}\right|}{\left|A_{l}^{j,t_{3},t_{4}}\right|}$falls in the range [0, 0.5] (first category), then either$2A_{l}^{j,t_{1},t_{2}}+A_{l}^{j,t_{3},t_{4}}>0$and$2A_{l}^{j,t_{1},t_{2}}-A_{l}^{j,t_{3},t_{4}}<0$(upper cone) or$2A_{l}^{j,t_{1},t_{2}}+A_{l}^{j,t_{3},t_{4}}<0$and$2A_{l}^{j,t_{1},t_{2}}-A_{l}^{j,t_{3},t_{4}}>0$(lower cone). Points within this region are assigned a value of 1, while those outside are assigned a value of 0, yielding a binary map for this category. However, for differentiability and to smooth the boundary between categories, we employ the sigmoid function:

**Fig. 2.**
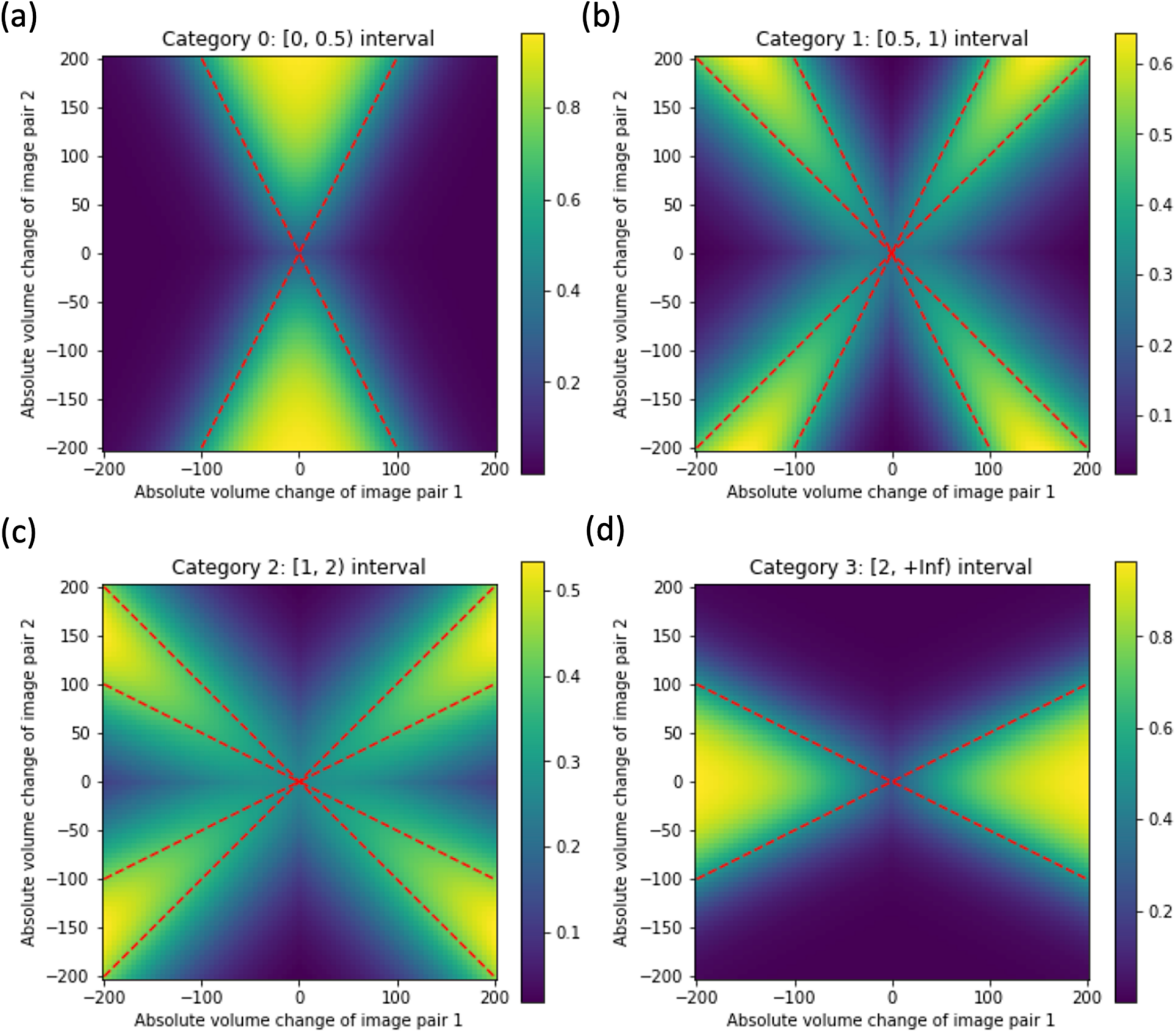
Functions used to map a pair of absolute volume change values,$A_{l}^{j,t_{1},t_{2}}$and$A_{l}^{j,t_{3},t_{4}}$, corresponding to two image pairs$\left(I^{j,\;t_{1}},I^{j,\;t_{2}}\right)$and$\left(I^{j,\;t_{3}},I^{j,\;t_{4}}\right)$, into a vector of probability values, corresponding to four categories (one-hot vector), during the computation of the Relative Interscan Interval (RISI) loss. The colors, ranging from navy blue to yellow, indicate the probability of the absolute attention volume changes in the two pairs of images belonging to each category. The red dashed lines represent the exact boundaries for each category, which are transformed into differentiable functions using sigmoid functions. Further details can be found inSection 2.4.

$$\begin{array}{l} P_{\left[0,0.5\right]}^{I}=P_{\left[0.0.5\right]}(A_{I}^{j,t_{1},t_{2}},A_{I}^{j,t_{3},t_{4}})=\max(\sigma_{\alpha }(2A_{I}^{j,t_{1},t_{2}}+A_{I}^{j,t_{3},t_{4}})\\ \;\;\;\;\;\;\;\;\;\;\;\;\;\;\;\;\cdot \sigma_{\alpha }(-2A_{I}^{j,t_{1},t_{2}}+A_{I}^{j,t_{3},t_{4}}),\sigma_{\alpha }(-2A_{I}^{j,t_{1},t_{2}}-A_{I}^{j,t_{3},t_{4}})\\ \;\;\;\;\;\;\;\;\;\;\;\;\;\;\;\cdot \sigma_{\alpha }(2A_{I}^{j,t_{1},t_{2}}-A_{I}^{j,t_{3},t_{4}})) \end{array}$$ (8)

Here$\sigma_{\alpha }\left(x\right)=1/(1+e^{-\alpha x})$withαas a scale parameter. The formulation for the remaining three categories is explained in detail inSupplementary Section S1. The resultant smooth map for category [0, 0.5] is plotted in Figure 2-(a), with corresponding maps for the other three categories in Figure 2-(b-d). The hyperparameterαgoverns the softness of the decision boundary.

Once$A_{l}^{j,t_{1},t_{2}}$and$A_{l}^{j,t_{3},t_{4}}$are mapped to a one-hot vector, the cross-entropy loss is applied:

$$\begin{array}{c} l_{RISI}^{l}=l_{CE}\left(\left[P_{\left[0,0.5\right]}^{l},P_{\left[0.5,1\right]}^{l},P_{\left[1,2\right]}^{l},P_{\left[2,\text{Inf}\right]}^{l}\right]\right). \end{array}$$ (9)

The overall RISI loss is computed as the sum of the individual losses for shrinkage and expansion regions:

$$\begin{array}{c} l_{RISI}=l_{RISI}^{SHR}+l_{RISI}^{EXP}. \end{array}$$ (10)

In the inference stage, there is no need to compute the STO and RISI losses, and only a single image pair$\left(I^{j,t_{1}},I^{j,t_{2}}\right)$is input to a single branch of the RDA network to generate a pooled attention area change$A_{\text{pooled}}^{j,t_{1},t_{2}}$.$A_{\text{pooled}}^{j,t_{1},t_{2}}$is calculated as a pooled of the volume of the shrinkage area and the expansion area in the corresponding attention maps:

$$\begin{array}{c} A_{\text{pooled}}^{j,t_{1},t_{2}}=A_{SHR}^{j,t_{1},t_{2}}-\;\;A_{EXP}^{j,t_{1},t_{2}}. \end{array}$$ (11)

As a longitudinal volume change measure,$A_{\text{pooled}}^{j,t_{1},t_{2}}$serves as a biomarker for neurodegeneration analysis in the RDA model for a single image pair.

### 2.4 Evaluation metrics

Four models are compared inSection 3. DBM method, ALOHA (Das et al., 2012), and DeepAtrophy (Dong et al., 2021) were selected as baseline models, to which RDA is compared. In addition, to test the effectiveness of the RISI loss, an ablation experiment was conducted where RDA was trained without the RISI loss, denoted RDA_STO_only_. In all compared models, the same train/evaluation/test split and the same image pairs were selected in the test stage.

In three models, ALOHA, RDA_STO_only_, and RDA, the rate of volumetric change in regions of interest (referred to as atrophy rate, although its calculation differs in models, described as below) was computed as a measure of disease progression rate. In ALOHA, atrophy rate was calculated as the difference in hippocampal volume (segmented by ASHS-T1) between the baseline and follow-up scans, divided by the baseline hippocampal volume (positive atrophy rate corresponds to hippocampal volume loss). In RDA_STO_only_and RDA, the pooled attention area change measure$A_{\text{pooled}}^{j,t_{1},t_{2}}$was computed, as described above, and used to represent atrophy rate. Analogous to ALOHA, a positive value of$A_{\text{pooled}}^{j,t_{1},t_{2}}$corresponds to volume loss in the shrinking ROIs and volume increase in the expansion ROIs. In DeepAtrophy, Predicted Interscan Interval (PII), as a measurement of predicted brain aging in comparison with actual interscan interval (assuming the same rate of disease and/or age progression for all individuals), was extracted as a measurement of the total amount of brain change (progression) in the MTL area. In addition, a single score Predicted-to-Actual Interscan Interval Rate (PAIIR) for each longitudinal image pair was derived as a surrogate biomarker of the rate of disease progression (Dong et al., 2021), and was compared with the other four methods. PAIIR is expected to be equal to 1 in the A- CU group, and larger with increasing clinical stages of AD.

#### 2.4.1 Scan Temporal Order (STO) and Relative Interscan Interval (RISI) inference accuracy

The first set of evaluation metrics focuses on the ability of the four models to correctly infer temporal information from longitudinal scans that are in arbitrary temporal order. These evaluation metrics, denoted “STO accuracy” and “RISI accuracy,” correspond to the STO and RISI losses used to train DeepAtrophy and RDA. They are evaluated on the test set, that is, participants whose scans were unseen by the models during training. STO accuracy is measured as the fraction of scan pairs for which the sign of the change measurement output by the model (PII for DeepAtrophy, hippocampal volume change for ALOHA, pooled attention area change$A_{\text{pooled}}^{j,t_{1},t_{2}}$for RDA variants) matched the sign of the true interscan interval. This accuracy measure is analogous to the ratio of hippocampal “growers” evaluated bySankar et al. (2017).

RISI accuracy is calculated as the fraction of experiments in which two pairs of scans from the same subject were input to a model, and the absolute value of the change measurement for the scan pair with a longer interscan interval was greater than the absolute value of the change measurement for the scan pair with a shorter interscan interval. Only subjects from the test set with at least three scans were selected, and the shorter interscan interval must fall within the longer one, as per the RISI training input conditions inSection 2.3.

Receiver operating characteristics (ROC) curve and the area under ROC (AUC) were reported for both STO accuracy and RISI accuracy using four methods, and DeLong’s test (Delong et al., 2012) with the “pROC” R package (Robin et al., 2011) was used to determine the significance of difference in AUC between all three deep learning methods and ALOHA.

#### 2.4.2 Group differences in rates of disease progression

To assess the effectiveness of various measurements in detecting differences in rates of disease progression between individuals on different stages of the AD continuum, we compared the effect sizes for group comparisons between three A+ groups (A+ CU, A+ eMCI, A+ lMCI) and the A- CU group using PAIIR for DeepAtrophy and annualized ROI volume change rate (atrophy rate) for other methods. To simulate different longitudinal study scenarios, this group difference comparison was conducted separately by selecting for each subject either all follow-up scans in the 180-to-400-day window from the baseline scan or in the 400- to 800-day window from the baseline scan. The test set was reduced to 250 subjects for the 400-day experiments and 226 subjects for the 800-day experiments, using the same subjects and scans for all methods.

When conducting this group difference analysis, a single measurement was derived for each subject and method, regardless of the number of scans available. For subjects with only two scans, the annualized ROI volume change rate was calculated as the ratio of the volume difference divided by the time elapsed between scans for all models, except DeepAtrophy. This value was then normalized by dividing it by the volume of the first scan to account for variations in brain size, ensuring that comparisons of absolute volume differences were not misleading. For ALOHA, the first scan’s volume corresponds to the hippocampus segmentation volume, while for RDA variants, the first scan’s volume is the sum of both shrinkage and expansion regions$\left(A_{SHR}^{j,t_{1},t_{2}}\;\;+\;\;A_{EXP}^{j,t_{1},t_{2}}\right)$, as both regions contribute to atrophy calculations. For subjects with multiple scans, a single measurement was used to represent an overall progression rate for each method. For DeepAtrophy, a linear model was fit using PII values from all available scans, and the slope was taken as the summary PAIIR for the subject. For other methods, a linear model was fit to volumetric measurements using scan pairs, and the slope represents an annualized volume change with a unit of mm^3^. This slope was divided by the baseline volume (hippocampus segmentation volume for ALOHA, and the sum of shrinkage and expansion areas for RDA variants) to get an annualized volume change rate, which was then corrected for age based on a group-wise linear model. An unpaired one-sided Wilcoxon test (Bauer, 1972) was then conducted to compare disease progression measurements between the A+ groups (A+ CU, A+ eMCI, A+ lMCI) and the normal group (A- CU). Areas Under the Curve (AUC) are reported to quantify the magnitude of the difference between each disease group and the A- CU group in terms of their means, relative to the variability observed in the data.

#### 2.4.3 Attention heatmaps of Regional Deep Atrophy

The RDA model predicted an attention map for each longitudinal scan pair in the MTL to show areas of “shrinkage,” “expansion,” and the background. To summarize the attention map and evaluate RDA’s explanation of regions of progression, an average heatmap was created from the shrinkage and expansion areas across all longitudinal pairs in the test set. To bring all attention maps to an average heatmap space, unbiased population templates of bilateral MTL provided by ASHS-T1 (Xie, Wisse, et al., 2019) were used as a reference, and all the fixed images in RDA in the test set were warped to the template space through deformable registration using the package*greedy*(Yushkevich et al., 2016). The attention maps of each scan were also warped to the template space using the corresponding deformation fields. In the template space, a heatmap for each voxel was calculated as the frequency of it being identified as shrinkage/expansion area divided by the total number of test examples. For instance, the attention heatmap for shrinkage area was calculated as

$$\begin{array}{c} H_{SHR}\left(x\right)=\frac{\sum_{\left\{k,a,b\right\}}\psi^{k,t_{a},t_{b}}\left(S\left(SHRk,t_{a},t_{b}\left(x\right)\right)\right)}{\sum_{\left\{k,a,b\right\}}\psi^{k,t_{a},t_{b}}\left(I^{k,t_{a},t_{b}}\left(x\right)\right)}, \end{array}$$ (12)

wherekis a subject in the test set,aandbare scan times from all available scan pairs for subjectk, is the shrinkage area of the fixed image,$k,S{(}_{SHR}^{k,t_{a},t_{b}}(x))$represents the fixed image area, and$\psi^{k,t_{a},t_{b}}$is the deformable transformation of this image area to the template space. A higher value in the shrinkage attention heatmap indicates the tissue area is strongly linked to atrophy according to RDA’s prediction. The peripheral areas in the template space may have fewer registered voxels compared with central areas, as the test images were cropped around the MTL segmentation during the data preparation step, and the deformable registration to the template space may not cover the entire template area.

### 2.5 Implementation details

The RDA model was trained on a single NVidia RTX A5000 GPU computer using 6,154 image pairs for RDA_STO_only_training, and 71,350 two-image pairs for RISI loss in RDA and DeepAtrophy training. For RDA and DeepAtrophy, STO and RISI losses are optimized simultaneously for 40 epochs. The learning rate was set to$1\times 10^{-5}$. Data augmentation applied to the deep learning pipeline included intensity normalization, random cropping centered on the MTL, random flipping, random rotation, and random image patch erasing. Each pair of longitudinal images underwent the same transformation (for example, rotation in the same angle, cropping at the same position) to ensure a voxel-wise match. Alignment was not applied to two image pairs from the same subjects during RISI training for RDA. For RDA_STO_only_and RDA, intensity normalization, random cropping, random flipping, and random rotation were used. Details of training parameters, such as batch size, and the number of training images can be referred to inSupplementary Section S2.

The hyperparameters in our model were set without extensive tuning to show the feasibility of RDA. In RDA, the SoftMax function with a temperature parameter of 100 was applied to the predicted probabilities of three regions to produce a 0 or 1 prediction for each voxel. Other hyperparameters such as the weight between the STO and RISI loss (1 and 1) and the number of classification categories (4) were set the same as in DeepAtrophy.

## 3 Results

### 3.1 Scan Temporal Order (STO) inference accuracy

**Fig. 3.**
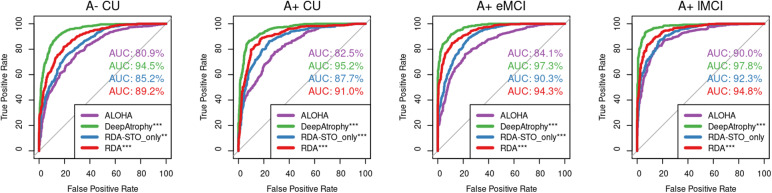
Area under the receiver operating characteristic (ROC) curve (AUC) for the scan temporal order (STO) inference experiments for all four models. This figure uses the same data as inTable 3. Greater AUC for DeepAtrophy, RDA, and RDA_STO_only_indicates greater accuracy in inferring the temporal order of scans. In each subplot, the DeLong’s test was conducted to compare each algorithm with the baseline algorithm ALOHA, and the results are shown for each comparison. ALOHA = Automatic Longitudinal Hippocampal Atrophy software/package; RDA = Regional Deep Atrophy; RISI = Relative Interscan Interval; A+ /A- = β-amyloid positive/negative; CU = cognitively unimpaired older adults; eMCI = early mild cognitive impairment; lMCI = late mild cognitive impairment; *p < 0.05; **p < 0.01; ***p < 0.001.

Table 3reports the mean STO accuracy for all five evaluated models on the test set.Figure 3further plots the receiver operating characteristic (ROC) curves for STO accuracy for the five methods and reports area under the ROC curve (AUC). Low STO accuracy for a given method may reflect the contribution of nonsystematic factors to the change measurement or the lack of systematic changes over time. For all methods, we expect STO accuracy to be lower for scan pairs with shorter interscan intervals and for individuals with less advanced disease, since in both of these cases, less systematic change is expected. Observations inFigure 3match this prediction: for all models, STO accuracy increases with more severe disease. Models that explicitly optimize STO accuracy (DeepAtrophy, RDA_STO_only_, and RDA) during training have significantly larger AUC (deLong’s test) compared with ALOHA. DeepAtrophy exhibits superior performance across all disease stage groups and overall. This could be attributed to DeepAtrophy’s greater flexibility in model training compared with RDA and RDA_STO_only_.

**Table 3.** Average accuracy for five models in inferring scan temporal order of the same subject scan pairs input in arbitrary order (STO Accuracy).

	A- CU	A+ CU	A+ eMCI	A+ lMCI	Average
ALOHA	73.6%	74.7%	77.3%	82.9%	76.6%
Deep Atrophy	**87.6%**	**87.7%**	**89.8%**	**93.2%**	**89.3%**
RDA _STO_only_	74.4%	79.4%	81.7%	84.0%	79.4%
RDA	77.3%	81.9%	84.7%	86.9%	82.3%

For all methods, lower accuracies in less impaired groups are expected because these groups undergo less neurodegeneration. Bold indicates the highest accuracy for each diagnostic group. ALOHA = Automatic Longitudinal Hippocampal Atrophy software/package; RDA = Regional Deep Atrophy; RISI = Relative Interscan Interval; A+ /A- = β-amyloid positive/negative; CU = cognitively unimpaired older adults; eMCI = early mild cognitive impairment; lMCI = late mild cognitive impairment.

### 3.2 Relative Interscan Interval (RISI) inference accuracy

Table 4compares the mean accuracy of four models in determining longer/shorter interscan interval (RISI accuracy) between two image pairs of the same subject. As with STO accuracy, we expect RISI accuracy to be higher for groups with more advanced disease because the amount of underlying systematic changes in these groups is greater. Models that explicitly optimize RISI accuracy (DeepAtrophy and RDA) during training have higher RISI accuracy compared with other models. RDA and DeepAtrophy achieve comparable RISI accuracy despite their different neural network architectures. Although only explicitly trained on STO loss, RDA_STO_only_has improved RISI accuracy compared with ALOHA (79.5% vs. 75.6%), demonstrating complementary nature of STO and RISI loss in capturing time-related information in longitudinal volume change analysis.

**Table 4.** Comparison of Relative Interscan Interval (RISI) accuracy for four models.

	A- CU	A+ CU	A+ eMCI	A+ lMCI	Average
ALOHA	69.4%	72.7%	79.4%	82.7%	75.6%
Deep Atrophy	**81.1%**	**88.7%**	86.9%	**90.3%**	**86.1%**
RDA _STO_only_	74.8%	76.1%	82.8%	85.1%	79.5%
RDA	79.2%	83.2%	**88.7%**	88.5%	84.7%

For two pairs of scans from the same subject, with the interscan interval of one scan pair strictly covers the other scan pair, we compare the accuracy of correctly identifying which scan pair has a longer interscan interval. For all methods, lower accuracies in less impaired groups are expected because less biological change happens in these subjects. Bold indicates the highest accuracy for each diagnostic group. ALOHA = Automatic Longitudinal Hippocampal Atrophy software/package; RDA = Regional Deep Atrophy; RISI = Relative Interscan Interval; A+ /A- = β-amyloid positive/negative; CU = cognitively unimpaired adults; eMCI = early mild cognitive impairment; lMCI = late mild cognitive impairment.

**Fig. 4.**
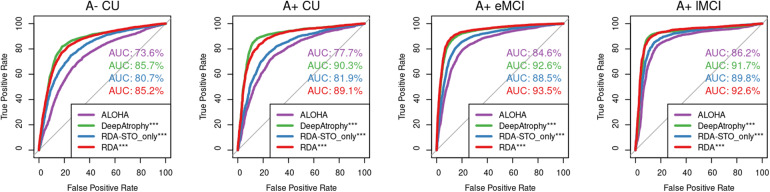
Area under the receiver operating characteristic (ROC) curve (AUC) for relative interscan interval (RISI) accuracy experiments for all four models. This figure uses the same data as inTable 4. Greater AUC for DeepAtrophy, RDA, and RDA_STO_only_indicates greater accuracy in inferring longer/shorter interscan interval for image pairs. In each subplot, the DeLong’s test was conducted to compare each algorithm with the baseline algorithm ALOHA, and the results are shown for each comparison. ALOHA = Automatic Longitudinal Hippocampal Atrophy software/package; RDA = Regional Deep Atrophy; RISI = Relative Interscan Interval; A + /A- = β-amyloid positive/negative; CU = cognitively unimpaired adults; eMCI = early mild cognitive impairment; lMCI = late mild cognitive impairment; *p < 0.05; **p < 0.01; ***p < 0.001.

Figure 4shows an AUC curve of RISI accuracy of different disease stages as depicted inTable 4. For all models, the accuracy of both RISI and STO (Tables 3and4) has an increasing trend as disease severity go from A- CU (no disease) to A+ lMCI (most severe in our dataset). This outcome is predictable since as the disease worsens, the neurodegeneration is more pronounced in change detection compared with scanning noise.

### 3.3 Visualizing disease progression in individual subjects

InFigure 5, we plot the disease progression measurements of the four models for individual subjects over all scan times. For each subject and method, the change in measurement (PII for DeepAtrophy, hippocampal volume change for ALOHA, pooled attention area change for RDA & RDA_STO_only_) from the baseline scan to each follow-up scan is displayed. The expected trend is to increase over time for PII, and to decrease over time for other methods (representing shrinkage of hippocampus in ALOHA, or combined shrinkage in shrinkage areas and expansion in expansion areas in RDA variants). The relationship between brain structural progression and time is typically considered to be exponential (Jedynak et al., 2012). However, in practice, some studies also simplify this model as a linear relationship (Cash et al., 2015;Das et al., 2012), since annual atrophy rates for normal aging elderly and Alzheimer’s disease patients are relatively small (0.5%–2% per year).

**Fig. 5.**
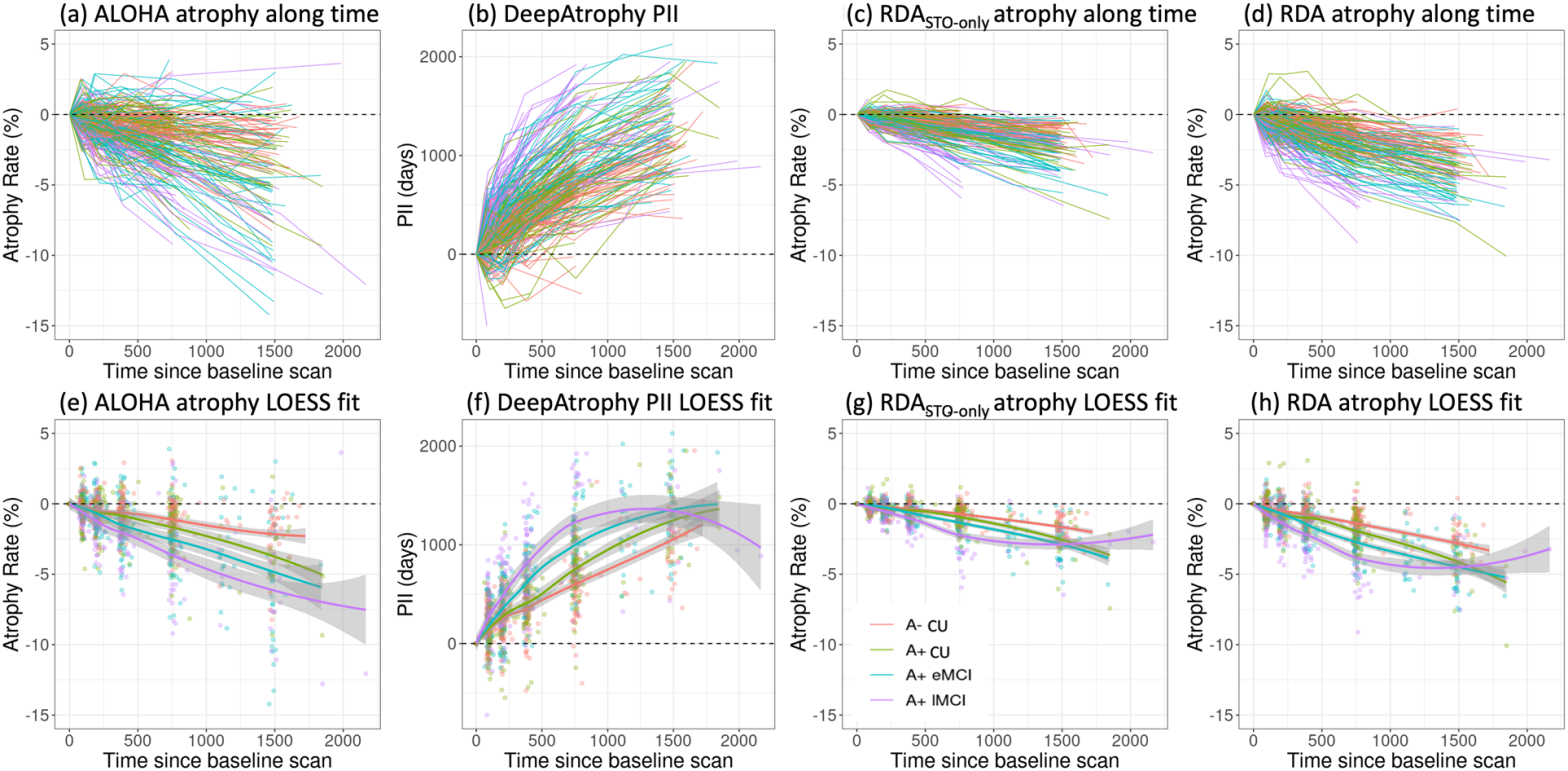
Comparison of (a, c, d) ALOHA, RDA_STO_only_, and RDA and volume change of different disease stages for individual subjects for all available scans; (b) DeepAtrophy Predicted Interscan Interval (PII). (e, f, g, h) are corresponding nonparametric fit and confidence interval (gray area) of each method. For DeepAtrophy, PII is expected to be positive since the baseline scan. For all other methods, atrophy rate is expected to be negative since the baseline scan. ALOHA = Automatic Longitudinal Hippocampal Atrophy software/package; RDA = Regional Deep Atrophy; RISI = Relative Interscan Interval; A+/A- = β-amyloid positive/negative; CU = cognitively unimpaired adults; eMCI = early mild cognitive impairment; lMCI = late mild cognitive impairment.

To assess the correlation between progression and time, we employed a nonparametric LOcal regrESSion (LOESS) model (Cleveland & Loader, 1996) to all the measurements (illustrated in the bottom row ofFig. 5), expecting a sublinear relationship. Our findings reveal that ALOHA and models incorporating deformation fields generally exhibit linear relationships, displaying heightened sensitivity to time scale compared with other methods. For DeepAtrophy, RDA_STO_only_, and RDA, the portion of lines inFigure 5in the “wrong” half-plane (above the x-axis for RDA/ALOHA; below x-axis for DeepAtrophy) is low, particularly for longer interscan intervals, suggesting that the use of STO loss in training reduces incorrect temporal order prediction due to nonsystematic factors. Compared with RDA_STO_only_, RDA has a smoother and more linear relationship with time than along with improved group distinction, suggesting that integrating the RISI loss enhances the network’s comprehension of disease progression relative to time. However, the LOESS fit for the A+ lMCI group in all deep-learning models demonstrates a notable decrease in linearity, in contrast with ALOHA and with other clinical groups. This trend could be attributed to the relatively small but dominant samples in the A+ lMCI group for scans occurring 1,500–2,000 days apart, or it may indicate that deep learning models underestimate atrophy when the amount of atrophy is significant.

### 3.4 Group differences in rates of disease progression

**Fig. 6.**
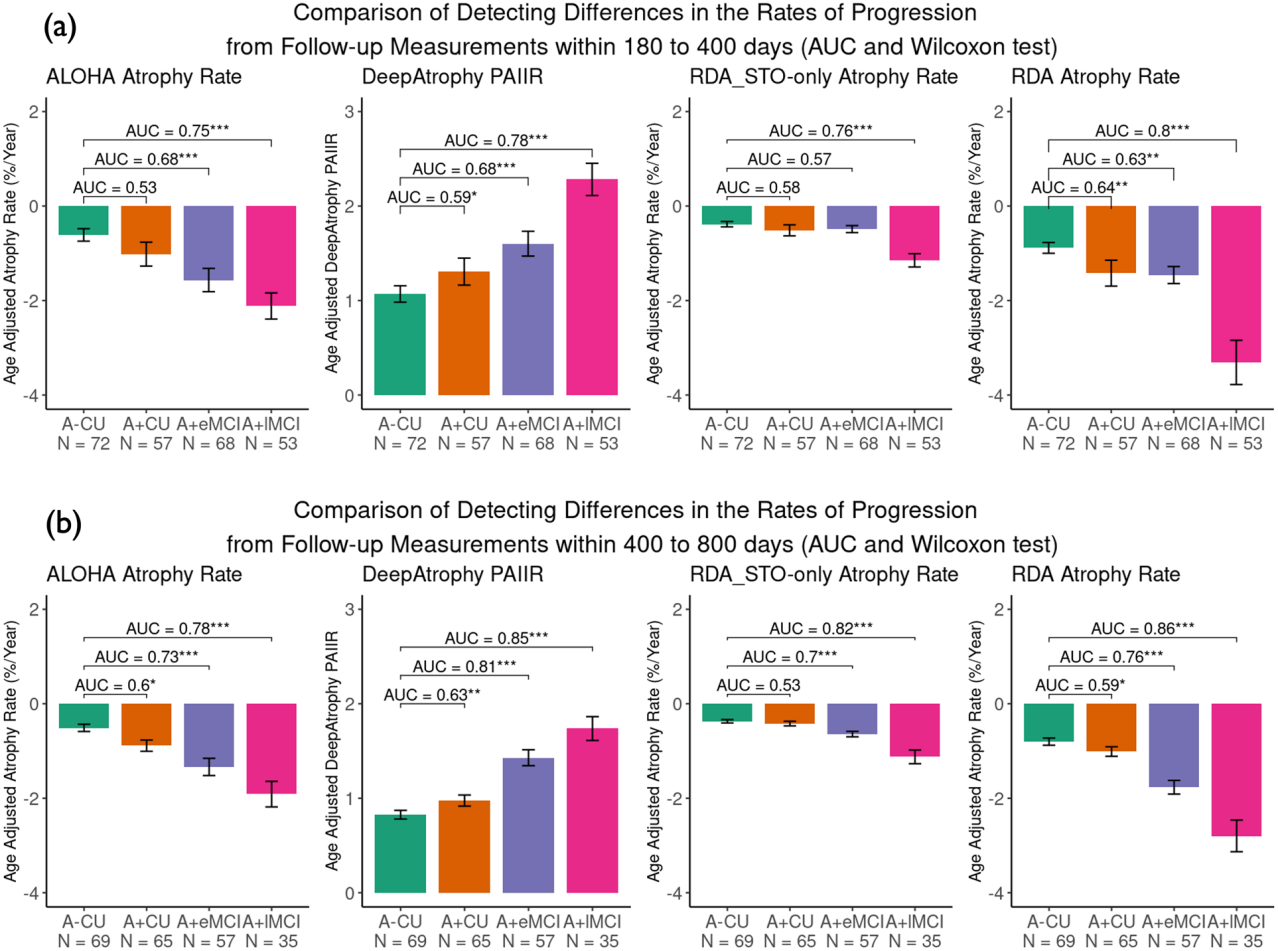
Comparison of four models to detect differences in rates of progression from follow-up measurements (a) within 180 to 400 days and (b) within 400 to 800 days. For DeepAtrophy, age-adjusted Predicted-to-Actual Interscan Interval Rate (PAIIR), and for the rest four methods, age-adjusted annualized atrophy rate was applied to differentiate groups. For RDA and RDA_STO_only_, age-adjusted annualized atrophy rate was calculated from pooled attention area change (by summarizing shrinkage and expansion regions, seeSection 2.5for details). In each subplot, the Area Under the Curve (AUC) and the Wilcoxon signed-rank test were conducted to compare each patient group with the control group, and the results were shown for each comparison. ALOHA = Automatic Longitudinal Hippocampal Atrophy software/package; RDA = Regional Deep Atrophy; RISI = Relative Interscan Interval; A+/A- = β-amyloid positive/negative; CU = cognitively unimpaired adults; eMCI = early mild cognitive impairment; lMCI = late mild cognitive impairment; *p < 0.05; **p < 0.01; ***p < 0.001.

Figure 6demonstrates the ability of different measurements (PAIIR for DeepAtrophy and annualized atrophy rate for others) to detect differences in rates of progression between the four disease groups over follow-up periods of 180–400 days (1 year) and 400–800 days (2 years). For almost all measurements, the sensitivity to disease stage is higher for scans with longer intervals (400–800 days) compared with shorter intervals (180–400 days). Within 400 days, RDA has the most significance in distinguishing between the normal control group (A- CU) and the preclinical AD group (A+ CU) (p = 0.0036, before False Discovery Rate correction), while DeepAtrophy is only marginally sensitive (p = 0.033), and ALOHA and RDA_STO_only_is not sensitive enough to detect differences with longitudinal MRI scans in such a short time. This highlights that RDA not only offers excellent interpretability, but also has the potential to detect subtle changes in a short amount of time between scans and to serve as a deep learning-based biomarker for longitudinal MRI measurements. Within 800 days, DeepAtrophy has the best significance in detecting difference in rates of progression at preclinical AD stage. AUC provides a different perspective for quantifying and interpreting differences in disease progression. Across all methods, higher AUCs are observed for longer scan intervals (400–800 days) and more advanced disease stages, indicating more pronounced changes in disease progression over time. RDA demonstrates relatively larger AUC in most differentiating stages, suggesting that RDA analysis may exhibit a greater disparity in the rate of disease progression.

### 3.5 Attention heatmaps of Regional Deep Atrophy

**Fig. 7.**
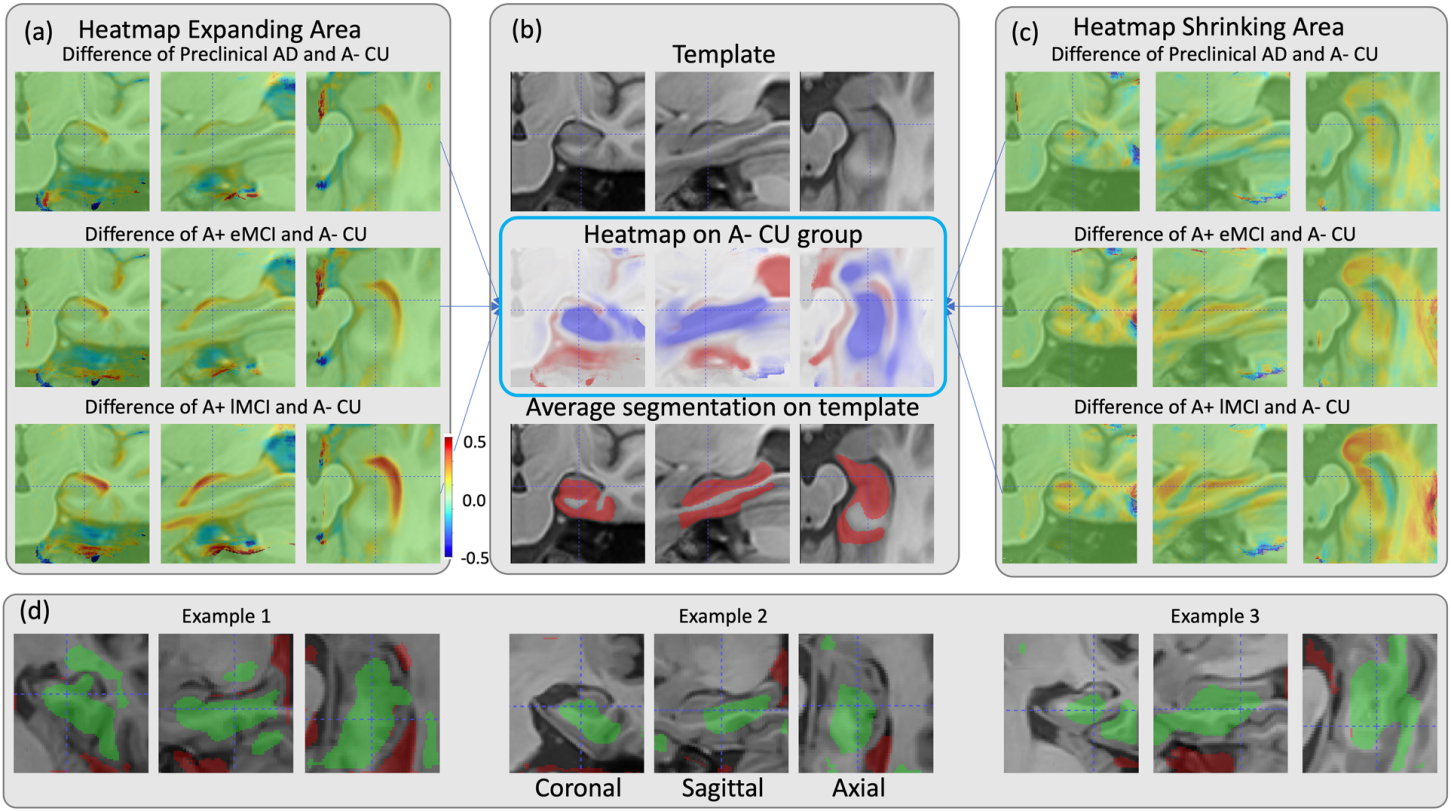
Regional Deep Atrophy (RDA) predictions depicted through attention maps of (b) averaged medial temporal lobe (MTL) in the template space of A- CU group, (a, c) the difference heatmap between all other diseased groups and the A- CU group, and (d) example attention maps as direct outputs of RDA. In panel (b), the MTL template, heatmap of shrinkage (in red) and expansion (in purple) areas, and the average MTL segmentation on the template (in red), are presented. In panels (a) and (c), areas with no change are denoted in green, while regions with more prominent heatmap (expansion for (a), and shrinkage for (c)) are depicted in hot colors (red), and lighter heatmap areas compared with the A- CU group are shown in cool colors (blue). For a single subject in panel (d), shrinkage areas are overlaid on MRI images in green, and expanding areas are overlaid in red (refer to color-printed version for details).

Figure 7displays the shrinkage and expansion regions identified by RDA through a heatmap of averaged MTLs on the template space of the A- CU group, the difference heatmap between all other diseased groups and the A- CU group, and some example attention maps directly generated by RDA. The heatmap reveals that the attention regions corresponding to shrinkage are concentrated in the gray matter of the MTL and white matter of the parahippocampal gyrus, while expansion regions are concentrated in the ventricles and other CSF regions. Despite the absence of anatomical information during training, RDA’s learned regions roughly correspond to anatomical structures associated with disease progression described in the literature (Braak & Braak, 1995;Platero et al., 2019). When comparing the differences among all diseased groups with the A- CU group, a progressively more prominent shrinkage area emerges in the anterior hippocampus, while expansion areas become more pronounced around the ventricles. The excessive changes observed in the peripheral area of the template are likely attributed to a bias resulting from the reduced representation of example images during the registration of individual attention maps to the template. InFigure 7(d), where the attention map of a single subject is displayed, the shrinkage and expansion areas are not contiguous. Background regions may indicate minimal structural change, or the biological change measurement is obscured by MRI noise and artifacts.

## 4 Discussion

In this study, we introduced a new deep learning model called Regional Deep Atrophy (RDA) that seeks to improve the interpretability of our earlier technique DeepAtrophy for estimating neurodegeneration in longitudinal MRI scans. The main motivation behind DeepAtrophy was to reduce the confounding effects of nonsystematic changes, such as MRI artifacts, on longitudinal change measures by using temporal inference loss functions that prime the algorithm to only detect time-related changes between image scans, hypothesizing that changes associated with the passing of time are mainly due to neurodegeneration. However, DeepAtrophy only provided a single summary measure of disease progression that lacks interpretability, making it difficult to determine what anatomical regions and what aspects of change (e.g., texture changes vs. boundary shifts) contribute to change measurements. RDA was designed to increase the interpretability of DeepAtrophy predictions and associate this prediction with a specific type of structural changes (growth/expansion) in the MTL area. RDA uses deformation fields from ALOHA as the registration backbone, and a segmentation-like network (namely 3D U-Net) to identify regions corresponding to shrinkage and expansion. Unlike DBM, which requires additional segmentation of ROIs for reporting atrophy rate measurements, RDA automatically detects regions in the scans that show longitudinal change, both shrinkage and expansion, eliminating the need for external segmentation in atrophy measurement. Results of DeepAtrophy and RDA were compared with the DBM model (ALOHA) as a representative baseline, based on prior comparisons byCash et al. (2015)andSankar et al. (2017)that demonstrated no significant differences among various state-of-the-art BSI and DBM methods. Adopting the same temporal information losses as DeepAtrophy, RDA reaches a similar accuracy and group differentiation ability as DeepAtrophy, while also generating attention maps related to longitudinal structural change. As such, RDA combines the benefits of DeepAtrophy (high temporal inference accuracy, group differentiation) and DBM (interpretability).

### 4.1 Regional Deep Atrophy achieves high temporal inference accuracies

Sources of change observed in longitudinal images can be classified into systematic and nonsystematic. Systematic changes include factors such as neurodegeneration, scanner update, and chronic ARIA induced by treatment, while nonsystematic change encompasses variations in patient hydration levels, head motion during scanning, MRI artifacts, and transient ARIA (Table 1). Conventional longitudinal measurements based on DBM do not explicitly differentiate between systematic and nonsystematic changes, which can lower their sensitivity to neurodegeneration. Both DeepAtrophy and RDA focus on extracting systematic changes from image pairs and thereby eliminating the influence of nonbiological and treatment-induced features in the dataset in order to isolate neurodegeneration. The STO loss and RISI loss were designed to achieve this objective. Clinically, high STO or RISI accuracy indicates that the detected neurodegeneration level surpasses noise from nonsystematic factors, and that the algorithm accurately identifies the chronological order of scans.

The findings reveal that among methods utilizing deformation fields (RDA, RDA_STO-only_, and ALOHA), RDA achieves the highest temporal inference accuracies, approximately 82.3% and 84.7%, respectively, for STO and RISI (Tables 3and4;Figs. 3and4). However, this accuracy is slightly lower than that of DeepAtrophy, which is 89.3% and 86.1%, respectively. This difference may be attributed to DeepAtrophy not restricting change measurement to deformation fields, affording it more flexibility, for example, to use texture information. Conversely, the registration-based method ALOHA demonstrates lower accuracy, at 76% for both STO and RISI. This suggests that simply applying deformation to anatomical ROIs, as is done in ALOHA and other DBM methods, is susceptible to confounds from nonsystematic sources.

To further examine the extent to which nonsystematic factors contribute to differences in STO accuracy between RDA and ALOHA, we studied the relationship between the STO accuracy between scan pairs and image similarity scores in a separate study. The underlying assumption is that low similarity scores are due to low image quality and presence of imaging artifacts in one or both of the time points. Results show that for both RDA and ALOHA, STO accuracy is higher with middle NCC similarities (0.75–0.90), and it drops when NCC scores are higher (close to 1) or very low (<0.25), although the absolute values of STO accuracy are higher for RDA compared with ALOHA. This may suggest that STO accuracy is easily confounded when either image pairs are very similar and not much progression can be observed (high NCC), or image pairs are not aligned at all (low NCC).

### 4.2 Regional Deep Atrophy-generated heatmaps correspond to anatomical structure

We relaxed ROIs from whole anatomical areas to automatically learned regions that are more sensitive to temporal information. Our hypothesis is that by training the model to detect temporal changes, it can focus on regions that are more related to time, and ignore random signals in the image that are not related to time, and thus provide sensitivity to progressive changes. Since some changes measured from registration methods may not be due to biological changes, but rather to noise, we infer that the learned attention map area is potentially more specific to biological changes in the brain. This hypothesis is supported by the observation that temporal inference accuracy generally increases with the severity of AD for all compared methods, indicating a positive correlation between biological changes in the brain and the ability of the models to detect these changes.

The attention maps generated by RDA are consistent with areas reported in previous literature to be sensitive to longitudinal change. AD-related brain degeneration typically starts in the MTL, including the hippocampus and entorhinal cortex, and gradually spreads to cerebral cortex and the whole brain (Braak & Braak, 1995;Hua et al., 2016;Mueller et al., 2005;Vemuri & Jack Jr, 2010). Hippocampus atrophy and ventricle expansion are commonly used as indicators of disease severity due to their close relationship with later cognitive functionality and higher consistency in tissue segmentation (Cash et al., 2015;Marinescu et al., 2019). The average attention heatmap of RDA highlights shrinkage areas of the hippocampus, amygdala, MTL cortex, and surrounding white matter, as well as an expanding area of lateral ventricle. These areas largely overlap with previously identified tissue change regions in the literature (Pettigrew et al., 2017;Wisse et al., 2014;Xie et al., 2020), as well as*ex vivo*3D analysis of tau spread in AD (Ravikumar et al., 2021). Example attention maps also show that RDA’s detected shrinkage areas are slightly distanced from the boundary between CSF and gray matter, whereas expanding areas appear to be tangent to it. This phenomenon could stem from tissue structure heterogeneity within the MTL, leading to uncertainty in the deformation field and introducing variation when detecting shrinkage areas in the attention map. By excluding signals from these regions compared with traditional anatomy-based analysis, RDA may exhibit increased sensitivity compared with ALOHA.

### 4.3 Regional Deep Atrophy-generated biomarker may be sensitive for early detection of AD

Measures of longitudinal neurodegeneration are a critical tool for studying disease progression and treatment response in Alzheimer’s disease and related dementias (ADRD), yet there is no ground truth against which these measures can be validated (Cash et al., 2015). Comparisons in rates of change between disease stages are a surrogate evaluation, premised on the hypothesis, confirmed in many prior studies (Jack et al., 2018), that individuals with more advanced disease undergo accelerated neurodegeneration. The ability to detect differences in the preclinical stage of AD is particularly relevant in the context of evaluating change detection methods, since individuals at this stage are thought to exhibit slower rates of neurodegeneration that may be masked by nonsystematic factors. We used bar plots to compare atrophy rates in different groups, with a focus on the preclinical AD group (A+ CU). Our results showed a significance (p = 0.0075) in RDA in differentiating the preclinical AD group and the A- CU group, even when using data from a 180–400-day follow-up window, suggesting that these methods could also be effective biomarkers in the context of a drug trial focused on preclinical AD. As deep learning is a fast-emerging field and these are preliminary results, we believe both DeepAtrophy and RDA have the potential to lead to more sensitive disease progression biomarkers in preclinical AD.

### 4.4 Explainable and longitudinal deep learning for AD biomarker studies

Most DL-based analyses of longitudinal or cross-sectional MRI in AD are more focused on automatic diagnosis (Hao et al., 2020;Lee et al., 2019), prediction of future disease progression (Ghazi et al., 2019;Gibson et al., 2018;Q. Li et al., 2019), or cognitive score prediction (Lu et al., 2021;Nguyen et al., 2020). In one of the recent longitudinal MRI challenges, TADPOLE challenge (Marinescu et al., 2019), three objectives were defined for longitudinal image prediction: AD diagnosis, cognitive score, and ventricle volume for each subject. In this challenge, most highly ranked teams are based on XGBoost (Marinescu et al., 2020) and statistical regression models (Aksman et al., 2019). However, these objectives are distinct from the goal of our paper, which is to develop a biomarker based on longitudinal MRI that is sensitive to differences in disease progression.

In addition, there is limited research on quantifying longitudinal atrophy in Alzheimer’s disease using interpretable deep learning methods. Most previous studies (Hosseini-Asl et al., 2016;Yue et al., 2019) have focused on classifying disease stages (e.g., AD vs. NC) through deep learning models. Of them, some models incorporate interpreting techniques, such as self-attention mechanisms (Jin et al., 2020), to the classification task. These models analyze whole brain MRI images (Jin et al., 2020;Lee et al., 2019;Qiu et al., 2022;Tang et al., 2019) and generate heatmaps for each individual to highlight regions associated with AD. Most models show large areas and high probabilities (or p-values) in subjects with severe AD compared with cognitively normal individuals. Some models (Eitel et al., 2019;Zhang et al., 2022) ignore structural boundaries and learn a probability score for each voxel in the brain, while others (Zhu et al., 2022) use segmentations of different brain regions and learn the probability that a brain region is associated with the onset of AD. Our model focuses on the early onset of AD and learns to identify regions related to AD progression longitudinally. Our study is also unique in that it learns both shrinkage and expansion areas without overlap, that may both be associated with AD development. In the future, we can improve our analysis by using higher resolution images and create a probability map for each subject instead of a nearly binary mask.

### 4.5 Limitations and future work

RDA identifies anatomical regions around the hippocampus with accelerated structural change, including not only atrophy in white matter and gray matter, but also expansion in CSF and ventricles. One limitation of the model is that it only qualitatively shows heatmaps, but does not have a way to evaluate them quantitatively. Another limitation is that the impact of smoothing parameters used by deformable registration in ALOHA on RDA performance and salience heatmaps remains ambiguous. To explore this, we could conduct an analysis utilizing different global smoothing parameters as well as tissue-weighted smoothing techniques (Draganski et al., 2011;Fiford et al., 2018).

In training the network to infer the magnitude of interscan intervals (RISI loss), initially, we considered formulating the model as a regression problem, where the network directly predicts interscan interval ratios and compares them with the true values. However, practical implementation revealed this approach to be unfeasible. Therefore, we opted to transform the problem from regression to classification. While this conversion simplified the problem, it also resulted in potential loss of information in the prediction.

Due to the time-consuming nature of manual image quality control, we implemented an automatic method using a threshold for SSIM scores. However, further investigation suggests that this method may also exclude scan pairs with significant anatomical deformations, which are common in subjects in the later stages of AD. This exclusion likely contributes to the limited number of A+ lMCI groups in our dataset, especially when images are scanned further apart in time, and may explain the observed drop in the nonparametric fit of progression over time for the A+ lMCI group, as shown inFigure 5.

In this paper, the MTL area was selected for longitudinal analysis primarily because it is the earliest site of AD onset and shows the most significant changes during AD progression. In future work, it would be intriguing to conduct analyses at the whole-brain level to explore AD progression patterns more comprehensively. Including more brain regions as input could enable RDA to learn more informative and less noisy representations. Our model focuses on using deep learning techniques to automatically extract features, utilizing basic components such as U-Net and ResNet. With the rapid advancement in deep learning, more sophisticated modules, such as attention modules (Vaswani et al., 2017) and Vision Transformer (Dosovitskiy et al., 2020;Liu et al., 2021), have proved to be effective in various medical image analysis applications. In the future, we will investigate the possibility of incorporating these advanced architectures into our model. To train our model effectively, a large dataset is required. Indeed, we excluded preclinical AD from training to maximize the number of such individuals available for comparing trained models. With the increasing availability of longitudinal preclinical AD datasets, such as A4 dataset (Sperling et al., 2014), we plan to leverage these datasets to improve model performance and evaluate the efficacy of our model across different datasets.

## 5 Conclusion

In this paper, we present Regional Deep Atrophy (RDA), a deep learning approach that can infer temporal information from same-subject longitudinal brain MRI scans. Like the earlier DeepAtrophy approach, the summary measures of change output by our method achieve excellent accuracy in ordering scans (newer/older) and interscan intervals (shorter/longer) compared with conventional registration-based methods, and can detect subtle differences in longitudinal change between groups at different stages of the AD continuum for both scans with 1 and 2 years intervals, including between cognitively unimpaired individuals with and without PET-based evidence of AD pathology. Unlike DeepAtrophy, which only provided a single summary measure of change and lacked interpretability, RDA produces a dense deformation field that describes the longitudinal change across the imaged region and it also generates attention maps corresponding to regions where the deformation field captures progressive changes (atrophy or expansion) in the brain, as opposed to spurious changes, such as those caused by MRI artifacts. The atrophy/expansion regions identified by RDA roughly align with anatomical knowledge, though appear biased toward central white matter regions. The combination of image registration and deep learning-based temporal inference in RDA creates an interpretable and accurate deep learning biomarker for quantifying disease progression in AD.

## Data and Code Availability

The data used in this study were obtained from the Alzheimer’s Disease Neuroimaging Initiative (ADNI) database (adni.loni.usc.edu). The ADNI data are publicly available to qualified researchers upon application and adherence to the ADNI Data Use Agreement. Access to the data requires registration and compliance with ADNI’s data use policies. Details on how to access the data can be found at the ADNI websitehttps://adni.loni.usc.edu/.

The code used for the analysis in this study is available at GitHub,https://github.com/MengjinDong/RegionalDeepAtrophy. Any additional custom code generated and used during the current study is available from the corresponding author upon reasonable request.

## Author Contributions

Mengjin Dong: Conceptualization, Methodology, Software, Validation, Formal analysis, Writing—original draft, Visualization. Long Xie: Methodology, Validation, Investigation, Data curation, Resources, Writing—review & editing, Supervision. Sandhitsu R. Das: Methodology, Investigation, Writing—review & editing. Laura E.M. Wisse: Investigation, Data curation. Robin deFlores: Investigation, Data curation. David A. Wolk: Methodology, Investigation, Funding acquisition. Paul A. Yushkevich: Conceptualization, Methodology, Formal analysis, Investigation, Writing—review & editing, Supervision, Project administration, Funding acquisition.

## Ethics Statement

Data used in this study were obtained from the Alzheimer’s Disease Neuroimaging Initiative (ADNI) database (adni.loni.usc.edu). The ADNI study was approved by the Institutional Review Boards (IRBs) of all participating institutions. Written informed consent was obtained from all participants or their authorized representatives before the collection of any data. The ADNI study adheres to ethical guidelines and principles set out by the Declaration of Helsinki.

## Declaration of Competing Interest

David A. Wolk has served as a paid consultant to Eli Lilly, GE Healthcare, and Qynapse. He serves on DSMBs for Functional Neuromodulation and Glaxo Smith Kline. He is a site investigator for a clinical trial sponsored by Biogen. Sandhitsu R. Das received consultation fees from Rancho Biosciences and Nia Therapeutics. Long Xie is a paid employee of Siemens Healthineers. The other authors have nothing to disclose.

## Acknowledgments

This work was supported by National Institute of Health (NIH) (Grants R01-AG069474, RF1-AG056014, R01-AG040271, P30 AG072979, R01-AG055005, R01-AG070592), and Foundation Philippe Chatrier. Data collection and sharing for this project was funded by the Alzheimer’s Disease Neuroimaging Initiative (ADNI) (National Institutes of Health Grant U01 AG024904) and DOD ADNI (Department of Defense award number W81XWH-12-2-0012). ADNI is funded by the National Institute on Aging, the National Institute of Biomedical Imaging and Bioengineering, and through generous contributions from the following: AbbVie, Alzheimer’s Association; Alzheimer’s Drug Discovery Foundation; Araclon Biotech; BioClinica, Inc.; Biogen; Bristol-Myers Squibb Company; CereSpir, Inc.; Cogstate; Eisai Inc.; Elan Pharmaceuticals, Inc.; Eli Lilly and Company; EuroImmun; F. Hoffmann-La Roche Ltd and its affiliated company Genentech, Inc.; Fujirebio; GE Health- care; IXICO Ltd.; Janssen Alzheimer Immunotherapy Research & Development, LLC.; Johnson & Johnson Pharmaceutical Research & Development LLC.; Lumosity; Lundbeck; Merck & Co., Inc.; Meso Scale Diagnostics, LLC.; NeuroRx Research; Neurotrack Technologies; Novartis Pharmaceuticals Corporation; Pfizer Inc.; Piramal Imaging; Servier; Takeda Pharmaceutical Company; and Transition Therapeutics. The Canadian Institutes of Health Research is providing funds to support ADNI clinical sites in Canada. Private sector contributions are facilitated by the Foundation for the National Institutes of Health (www.fnih.org). The grantee organization is the Northern California Institute for Research and Education, and the study is coordinated by the Alzheimer’s Therapeutic Research Institute at the University of Southern California. ADNI data are disseminated by the Laboratory for Neuro Imaging at the University of Southern California.

## Supplementary Materials

Supplementary material for this article is available with the online version here:https://doi.org/10.1162/imag_a_00294

## Supplementary Material

Supplementary Material
