# Supplementary material for: Regional deep atrophy: Using temporal information to automatically identify regions associated with Alzheimer’s disease progression from longitudinal MRI
Source: Imaging Neurosci (Camb). 2024 Sep 18;2:imag-2-00294. doi: 10.1162/imag_a_00294 (PMC12290704; doi:10.1162/imag_a_00294)
Supplement: Supplementary Material [file imag_a_00294-supp.pdf]

## S1. Formulation of Categorial Relative Interscan Interval (RISI) loss

To formulate Relative Interscan Interval (RISI) as a categorical loss, we need to construct a differentiable mapping between absolute volume change values, $A_l^{j,t_1,t_2}$ and $A_l^{j,t_3,t_4}$, and a vector of probability values (one-hot vector) corresponding to the discrete categories. Geometrically, the values $A_l^{j,t_1,t_2}$ and $A_l^{j,t_3,t_4}$ can be represented as a point in $\mathbb{R}^2$, and the four discrete categories can be represented as partitions of $\mathbb{R}^2$ by lines passing through the origin. For example, if $\frac{|A_l^{j,t_1,t_2}|}{|A_l^{j,t_3,t_4}|}$ is in the range [0, 0.5] (first category), then $2A_l^{j,t_1,t_2} + A_l^{j,t_3,t_4} > 0$ and $2A_l^{j,t_1,t_2} - A_l^{j,t_3,t_4} < 0$ (upper cone), or $2A_l^{j,t_1,t_2} + A_l^{j,t_3,t_4} < 0$ and $2A_l^{j,t_1,t_2} - A_l^{j,t_3,t_4} > 0$ (lower cone). The boundaries of the corresponding region are shown as dashed red lines in Figure 2-(a). Assigning points within this region value 1 and points outside of this region value 0, we would obtain a map that maps the pair $A_l^{j,t_1,t_2}$, $A_l^{j,t_3,t_4}$ to a near-probability value for this category (the probabilities of places close to the origin are low in all categories).

To simplify, let $B_{0.5,l}^j = 2A_l^{j,t_1,t_2} - A_l^{j,t_3,t_4}$ and $B_{-0.5,l}^j = 2A_l^{j,t_1,t_2} + A_l^{j,t_3,t_4}$ represent the lines passing through the origin with slopes of 0.5 and -0.5, respectively. A mathematical formulation of this area with Sigmoid smoothing can be represented as the maximum of two areas, with each area representing an overlapping of two half-planes:

$$P_{[0,0.5]}\left(A_l^{j,t_1,t_2}, A_l^{j,t_3,t_4}\right) = \max\left(\sigma_\alpha\left(B_{0.5,l}^j\right) * \sigma_\alpha\left(-B_{-0.5,l}^j\right), \sigma_\alpha\left(B_{-0.5,l}^j\right) * \sigma_\alpha\left(-B_{0.5,l}^j\right)\right)$$

where $\sigma_\alpha(x) = 1/(1 + e^{-\alpha x})$ and $\alpha$ is a scale parameter; $\sigma_\alpha\left(B_{0.5,l}^j\right) * \sigma_\alpha\left(-B_{-0.5,l}^j\right)$ and $\sigma_\alpha\left(B_{-0.5,l}^j\right) * \sigma_\alpha\left(-B_{0.5,l}^j\right)$ represent the upper and lower smooth maps with high probabilities for category [0, 0.5] in Figure 2-(a).

Similarly, let $B_{1,l}^j = A_l^{j,t_1,t_2} - A_l^{j,t_3,t_4}$, $B_{-1,l}^j = A_l^{j,t_1,t_2} + A_l^{j,t_3,t_4}$, $B_{2,l}^j = A_l^{j,t_1,t_2} - 2A_l^{j,t_3,t_4}$, and $B_{-2,l}^j = A_l^{j,t_1,t_2} + 2A_l^{j,t_3,t_4}$, representing all other decision boundaries, we have

$$P_{[0.5,1]}\left(A_l^{j,t_1,t_2}, A_l^{j,t_3,t_4}\right) = \max\left(\sigma_\alpha\left(B_{0.5,l}^j\right) * \sigma_\alpha\left(-B_{1,l}^j\right), \sigma_\alpha\left(-B_{-0.5,l}^j\right) * \sigma_\alpha\left(B_{-1,l}^j\right),\right.$$
$$\left.\sigma_\alpha\left(B_{-0.5,l}^j\right) * \sigma_\alpha\left(-B_{-1,l}^j\right), \sigma_\alpha\left(-B_{0.5,l}^j\right) * \sigma_\alpha\left(B_{1,l}^j\right)\right)$$

$$P_{[1,2]}\left(A_l^{j,t_1,t_2}, A_l^{j,t_3,t_4}\right) = \max\left(\sigma_\alpha\left(B_{2,l}^j\right) * \sigma_\alpha\left(-B_{1,l}^j\right), \sigma_\alpha\left(-B_{2,l}^j\right) * \sigma_\alpha\left(B_{-1,l}^j\right),\right.$$
$$\left.\sigma_\alpha\left(B_{-2,l}^j\right) * \sigma_\alpha\left(-B_{-1,l}^j\right), \sigma_\alpha\left(-B_{2,l}^j\right) * \sigma_\alpha\left(B_{1,l}^j\right)\right)$$

$$P_{[2,\infty]}\left(A_l^{j,t_1,t_2}, A_l^{j,t_3,t_4}\right) = \max\left(\sigma_\alpha\left(-B_{2,l}^j\right) * \sigma_\alpha\left(-B_{-2,l}^j\right), \sigma_\alpha\left(B_{-2,l}^j\right) * \sigma_\alpha\left(B_{2,l}^j\right)\right).$$

For categories 2 and 3, the regions within four cones have high probability, and a max function was used to combine the four regions. The corresponding maps for these three categories are plotted in Figure 2-(b-d).

## S2. Training configurations for different methods

Supplementary Table S1 shows the training configurations for the different methods. Training RDA requires multiple steps. For VoxelMorph and $RDA_{STO\text{-}only}$, only one longitudinal image pair is necessary for model training, and our dataset includes 6,154 training pairs. However, since RDA has more possible combinations of two longitudinal image pairs, the number of training images in the dataset increased to 71,350. Due to memory limitations, the batch size of RDA training is reduced to half that of VoxelMorph and $RDA_{STO\text{-}only}$. The table includes other parameters as well.

Supplementary Table S1. Training configurations for different methods. Abbreviations: # = number of; RDA = Regional Deep Atrophy; STO = Scan Temporal Order.

| | #training images | Batch-size | #epochs | #images/epoch | Training time (days) |
|---|---|---|---|---|---|
| DeepAtrophy | 71350 | 20 | 200 | 20*500 | 5 days |
| $RDA_{STO\text{-}only}$ | 6154 | 20 | 20 | 20*500 | 1 day |
| RDA | 71350 | 20 | 40 | 20*500 | 2 days |

## S3. MRI scanner information

All MRI scans were collected from the ADNI-2 and ADNI-GO studies, encompassing a total of 29 sites. The MRI imaging protocols used to acquire the 3T T1-weighted scans were previously described by Jack Jr et al. (2008) and Leow et al. (2006). The scans from the ADNI-2 and ADNI-GO datasets were obtained using MRI machines from multiple manufacturers, including Siemens, Philips, and General Electric (GE). The scanning protocols were standardized across different sites, with a repetition time (TR) of 2300 or 3000 ms, an inversion time (TI) of 853 to 900 ms, and a flip angle of 8-9 degrees. The whole-brain images were acquired at a resolution of 256x256x160.

## Reference:


Jack Jr, C. R., Bernstein, M. A., Fox, N. C., Thompson, P., Alexander, G., Harvey, D., Borowski, B., Britson, P. J., Whitwell, J. L., Ward, C., Dale, A. M., Felmlee, J. P., Gunter, J. L., Hill, D. L., Killiany, R., Schuff, N., Fox-Bosetti, S., Lin, C., Studholme, C., … Jr, C. (2008). The Alzheimer's Disease Neuroimaging Initiative (ADNI): MRI Methods. *Www. Interscience.Wiley.Com). JOURNAL OF MAGNETIC RESONANCE IMAGING*, *27*, 685–691. https://doi.org/10.1002/jmri.21049

Leow, A. D., Klunder, A. D., Jack, C. R., Toga, A. W., Dale, A. M., Bernstein, M. A., Britson, P. J., Gunter, J. L., Ward, C. P., Whitwell, J. L., Borowski, B. J., Fleisher, A. S., Fox, N. C., Harvey, D., Kornak, J., Schuff, N., Studholme, C., Alexander, G. E., Weiner, M. W., & Thompson, P. M. (2006). *Longitudinal stability of MRI for mapping brain change using tensor-based morphometry*. https://doi.org/10.1016/j.neuroimage.2005.12.013
